# Multi-classification deep neural networks for identification of fish species using camera captured images

**DOI:** 10.1371/journal.pone.0284992

**Published:** 2023-04-26

**Authors:** Hassaan Malik, Ahmad Naeem, Shahzad Hassan, Farman Ali, Rizwan Ali Naqvi, Dong Keon Yon

**Affiliations:** 1 Department of Computer Science, University of Management and Technology, Lahore, Pakistan; 2 Department of Computer Engineering, Bahria University Islamabad, Pakistan; 3 Department of Software, Sejong University, Seoul, South Korea; 4 Department of Intelligent Mechtronics Engineering, Sejong University, Seoul, South Korea; 5 Center for Digital Health, Medical Science Research Institute, Kyung Hee University Medical Center, Kyung Hee University College of Medicine, Seoul, South Korea; HITEC University, PAKISTAN

## Abstract

Regular monitoring of the number of various fish species in a variety of habitats is essential for marine conservation efforts and marine biology research. To address the shortcomings of existing manual underwater video fish sampling methods, a plethora of computer-based techniques are proposed. However, there is no perfect approach for the automated identification and categorizing of fish species. This is primarily due to the difficulties inherent in capturing underwater videos, such as ambient changes in luminance, fish camouflage, dynamic environments, watercolor, poor resolution, shape variation of moving fish, and tiny differences between certain fish species. This study has proposed a novel Fish Detection Network (FD_Net) for the detection of nine different types of fish species using a camera-captured image that is based on the improved YOLOv7 algorithm by exchanging Darknet53 for MobileNetv3 and depthwise separable convolution for 3 x 3 filter size in the augmented feature extraction network bottleneck attention module (BNAM). The mean average precision (mAP) is 14.29% higher than it was in the initial version of YOLOv7. The network that is utilized in the method for the extraction of features is an improved version of DenseNet-169, and the loss function is an Arcface Loss. Widening the receptive field and improving the capability of feature extraction are achieved by incorporating dilated convolution into the dense block, removing the max-pooling layer from the trunk, and incorporating the BNAM into the dense block of the DenseNet-169 neural network. The results of several experiments comparisons and ablation experiments demonstrate that our proposed FD_Net has a higher detection mAP than YOLOv3, YOLOv3-TL, YOLOv3-BL, YOLOv4, YOLOv5, Faster-RCNN, and the most recent YOLOv7 model, and is more accurate for target fish species detection tasks in complex environments.

## Introduction

As people’s living conditions continue to improve, aquatic goods have emerged as an increasingly essential source of protein. As a result, the aquaculture industry currently accounts for more than sixty percent of the world’s total production of these items [1–3]. The output of fish that has been raised in captivity constitutes a significant component of the entire aquaculture sector [4,5]. Aquaculture is becoming an increasingly important area of attention for several national governments as part of ongoing efforts to guarantee that their populations will always have ready access to adequate supplies of food [6]. The aquaculture industry is moving away from the more conventional approaches to artificial farming in favor of the more innovative and cutting-edge farming techniques that are collectively referred to as intelligent farming. This is a direct result of the rapid advancement of contemporary technology [7–9]. The use of computer vision technology (CVT) is essential to the development of intelligent aquaculture [10–12]. CVT is used extensively in fish farming [13] to detect, classify, identify, measure, and count fish [14,15]. This may be achieved by knowing the phenotypic traits of fish that live in underwater environments [16–18]. One of the most significant tasks in developing the sector of fish farming is to get reliable identification of individual fish [19].

Conventional methods for fish identification may use either the machine learning (ML) technique or manually determined criteria [20–22] to identify the species of fish being researched. In contrast, manual trait selection is inefficient, and characteristics derived from human experience are insufficient, leading to an unreliable degree of prediction [23–25]. As there is a growing interest in mechanized fish farming [26], simple species identification is no longer enough. Once the species of fish have been identified, each member of that species may be recognized by their unique identifier and be given a name. Because this makes it possible for individual fish to be more easily identified, it is of greater relevance in terms of directing the expansion of the fish farming industry. At this time, the bulk of solutions for fish individual recognition (FIR) [27] make use of DL models that are built on the framework for fish recognition. The framework for fish recognition consists of three processes: fish object detection, fish feature extraction, and fish feature comparison. The process of FIR is comprised of three stages: the image preprocessing stage [28], the feature extraction stage [29], and the classification [30] and identification step. The standard method for object recognition uses an algorithm that relies heavily on the manual selection technique for feature extraction [31–40]. This method chooses relevant features based on the subjectivity of humans. The selection of traits using this method is very subjective, inefficient, and prone to overlooking important attributes [41]. Classifiers like the Naive Bayes (NB) [36], Decision Tree (DT) [39], and Support Vector Machine (SVM) are often used by traditional methods. Their accuracy is restricted, and they are only useful for locating very small fish targets that have distinguishable characteristics [42–43].

Due to its ongoing progress, several academics have started using deep learning (DL) in their work on object identification algorithms [32–38]. DL can efficiently and automatically extract characteristics and learn new information. The object identification strategy that is based on DL thus has the potential to be used to fish for objects with large-scale attributes that are not very relevant. The R-CNN [15], the SSD [16], the YOLO series [18], and several other algorithms are included in object identification systems that are based on DL. The RCNN is a two-stage DL methodology that is based on candidate boxes [19–22]. It has a slow detection speed and cannot perform real-time detection. Both the SSD and the YOLO series algorithm are examples of single-stage, regression-based approaches to DL. Even if the SSD method is fast, a large number of the parameters have to be supplied manually, and the process of debugging is challenging. The YOLO series approach is well suited for identifying individual fish because of its rapid speed [27], high accuracy [28], simple debugging, and real-time detection capabilities [29].

In earlier studies, the identification of fish species in the open ocean relied on characteristics that had to be manually manufactured. The study that was carried out by Spam-pinato et al. [44] took into consideration the attributes of the form as well as the features of the texture. It is then possible to generate a three-dimensional representation of the fish by applying an affine transformation to each of the generated images and putting them through the same process. In the study [45], they were able to collect 66 different kinds of characteristics, some of which were the color, shape, and texture of different parts of the fish. They went on to design a hierarchical categorization scheme, which they referred to as the "Balance-Guaranteed Optimized Tree (BGOT)," intending to minimize the error accumulation problem to the greatest extent possible.

Both Vieira et al. [46] and Monczak et al. [47] discovered that their models were able to recognize fish calls with a longer duration and significant harmonics more accurately than calls with a shorter duration that was pulsed. Even though the detection and classification accuracy are not very high for every fish call or species, all acoustic studies of fish that have used automatic analysis methods have concluded that these methods provide the most efficient way to analyze long-term PAM datasets [48–50]. Using a kernel that was developed by Ruiz-Blais et al. [51] were able to identify the calls that are made by Jamaica weakfish (Cynoscion jamaicen-sis). The kernel was based on four call characteristics, and a call was only identified when all four call features surpassed their respective criteria, which the researchers had already specified in the beginning. In other words, for a call to be recognized, each of the four call attributes had to concurrently surpass their respective thresholds. Ricci et al. [52] applied a multi-kernel approach that was based on the two lowest harmonic frequencies of oyster toadfish sounds to identify oyster toadfish calls that were included within the recordings. This allowed them to successfully identify oyster toadfish calls. A CNN with three convolution layers was created by Salman et al. [53] to collect characteristics and feed them into popular classifiers for the identification of fish species. These common classifiers include SVM and K-nearest neighbors (KNN). Qin et al. [54] suggested the use of a CNN that had been trained from the ground up with the help of the Fish Recognition Ground-Truth dataset. This CNN was built using three layers of convolutional processing. PCANet [55] and LIN [56] are two examples of different kinds of deep architectures that were used by Sun et al. [57] in their attempt to extract characteristics from underwater photographs. A linear SVM classifier is used whenever classification work needs to be done. Using characteristics that were obtained from the activations of the seventh hidden layer of the pre-trained AlexNet model [58], the findings of Jager et al. [59] were input into a multi-class SVM classifier to classify the data.

Zhang et al. [60] came up with the AdvFish approach to find a solution to the problem of noisy backgrounds. They were able to do this by adding a new term in the loss function, which provided them with the capacity to fine-tune the ResNet50 model. Because of this term, the network can automatically discern between the areas with fish and those with more distracting background noise, allowing it to concentrate more of its attention on the areas with fish. In addition to this, it assists in the development of the network that differentiates the fish areas from other locations. The teacher-student paradigm was used by Pang et al. [61] to lessen the effect that interference had on the categorization of fish species. They were able to extract information regarding interference by reducing the difference between two distance matrices that had been separately constructed from a processed fish picture and a raw fish image. These matrices were formed from a fish image before any processing was done to them. They were able to trace the source of the interference as a direct result of this finding. An application of KL-divergence is performed on the distribution of the raw data to further cut down on the amount of noise that is present in the data. In recent years, advancements in computer vision have enabled a way for identifying fish movement that is both quick and nondestructive. This approach was developed to take advantage of these recent developments [62]. Labuguen et al. [63] continue the work that was done in the past [64] by describing an automated method to count fish. Image processing is used in this method to ascertain the pixel area that is occupied by the silhouette of each fish. To get started, a binarization and edge detection procedure is carried out, which involves placing a whole school of fish within a container that was designed to carry out such procedures. After that, they acquire the total number of fish as well as the average number of fish that are present in each picture frame by adding the area that is included inside each contour for each image frame. This allows them to determine the number of fish that are present in each image frame. Because of this, they can calculate not only the total number of fish but also the typical number of fish that may be found in each picture frame.

Using information obtained from underwater video recordings, Fabic et al. [65] devised an efficient approach for identifying fish, counting them, and establishing the species they belonged to. This approach depended on blob counting and form analysis to complete these tasks successfully. They deleted the coral from the background of the picture as part of an erasure procedure that they employed to assist in the identification of fish, and then they utilized canny edge detection to recover the fish outlines. The Zernike shape analysis was carried out to evaluate the degree to which the shapes of various fish species, particularly those belonging to the families Acanthuridae and Scaridae, are comparable to one another. This was done to determine whether or not there is a relationship between the shapes of these fish. Following the completion of the fish population estimate, the blob counting method was used to identify the total number of fish that were present in the population. In the end, due to the numerous shifts that took place in the image, they calculated the average fish count for each period by beginning with the counts that were present in each frame as their point of reference. The propagation of fish has turned into a roadblock in the path of the development of fish farming, and the counting method is one of the issues that come up at various points throughout the process of hatching fish eggs. The researchers that participated in the study [66] made use of a robotic eye camera to collect images of shrimp that were being raised on a shrimp farm for the goal of training the model. The information obtained from the photographs was analyzed and the results were categorized as having a "low density," "mid-density," or "high density" based on the number of shrimps that were visible in each picture. An improved model of the Mask Regional Convolutional Neural Network (also known as the Mask R-CNN) was created as a result of applying a parameter calibration technique to find the appropriate values for the network’s parameters. As a result of this, the enhanced Mask R-CNN model has the potential to attain an accuracy level of up to 97.48%. These days, marine ecologists perform research on the variety of life that may be found in underwater environments by making considerable use of underwater camera systems. These technologies are not harmful, do not result in any alterations to the environment that they are embedded in, and generate a significant amount of visual data that may be put to use at any point in time.

By using CNN and training it using a novel method that is based on incremental learning, Ben et al. [67] were able to classify live reef fish species in an unrestricted underwater environment. The method of gradual learning was crucial in bringing about this successful outcome. According to the results of the calculations that were carried out, the recommended method had an accuracy of 81.83% when applied to the LifeClef 2015 Fish benchmark dataset. Iqbal et al. [68] proposed the performance of research that would involve an efficient end-to-end CNN for the goal of classifying fish behavior into the normal and starving categories. This was done to categorize fish behavior. They evaluate the performance of CNN by modifying the number of fully connected (FC) layers and either utilizing or not utilizing the max-pooling method. The accuracy of the detection method is improved by 10% as a direct result of the incorporation of three FC layers in addition to the maximum pooling operation. According to the findings, the shallow architecture of the CNN model, which incorporates a max-pooling function with an increased number of FC layers, is capable of achieving an accuracy of 98% and displays promising performance. Roy et al. [69] proposed an improved version of YOLOV4 for fine-grain object detection. The proposed model is based on Spatial Pyramid Pooling (SPP), and a modified Path Aggregation Network (PANet) and they achieved an mAP of 96.29%. WilDect-YOLO is a DL-based automated high-performance detection model in the study [70] for real-time endangered wildlife identification. Using DenseNet-fused YOLOv4 and attaining an mAP of 96.20 percent, Roy et al. [71] proposed a one-of-a-kind real-time growth stage detection model that is capable of detecting stages with a high degree of occultation. Kaya et al. [72] proposed a CNN-based model named IsVoNet8 for the classification of fish species. They achieved a classification accuracy of 91.37%. LIBS and Raman’s spectroscopy were used in the study that Ren et al. [73] conducted to develop a novel method for the identification of fish species. The data from LIBS and Raman spectroscopies gathered from 13 different species of fish were used in conjunction with two different machine learning algorithms called SVM [74] and CNN [75,76] to construct classification models. The proposed CNN model achieves a maximum accuracy of classification of 96.2 percent.

The purpose of this research is to develop a deep learning (DL)-based methodology for the recognition of different species of fish. For this study, a proposed fish detection network (FD_Net) is based on the YOLOv7 algorithm by exchanging Darknet53 for MobileNetv3 and depthwise separable convolution for 3 x 3 filter size in the bottleneck attention module (BNAM) feature extraction network was designed for the detection of nine types of fish species i.e., gilt-head bream (GHB), red sea bream (RSB), sea bass (SB), red mullet (RM), horse mackerel (HM), black sea sprat (BSS), striped red mullet (SRM), trout (TRO), shrimp (SHR). The bounding box method and semantic segmentation are both viable detection methods included in this method. This methodology contributes to the preservation of aquatic fish species and facilitates their identification without the requirement of prior domain expertise. Additionally, this strategy assists in distinguishing the target fish from the rest of the school of fish. The primary objective is to construct a model that can detect and categorize the fish species that live in the water, one that makes use of trained architecture and computer vision algorithms that are capable of recognizing the fish species quickly and accurately. This research has major contributions which are discussed as follows:

1. The proposed FD_Net model is based on YOLOv7 by exchanging the feature extractor Darknet-53 with the MobileNet which is inspired by depth-wise separable convolution to detect nine types of fish species.
2. An improved iteration of DenseNet-169 serves as the foundation for the one-of-a-kind fish identification network known as FD_Net. The normal convolution is kept in the trunk of the network, while dilated convolution is introduced to the dense block of the network for feature extraction. Increasing the size of the receptive field makes it possible to acquire traits with greater specificity.
3. The FD_Net is trained and tested on a large-scale Dataset for fish Classification [44]. Extensive experiments were performed, as well as comparisons of the results with state-of-the-art approaches.
4. The FD_Net Loss function has been improved, which should result in greater overlap between the actual item and the predicted one.
5. Utilize the YOLOv7 technique to improve the accuracy of the model, which resulted in an improvement in the model’s accuracy as compared to the baseline model.

This study is further divided into four sections: Section 2 presents the recent literature. The material and methods of the present study are discussed in section 3. Section 4 contains the results and discussions. The conclusion and future work of this study is described in section 5.

## Materials and methods

**Fig 1:**
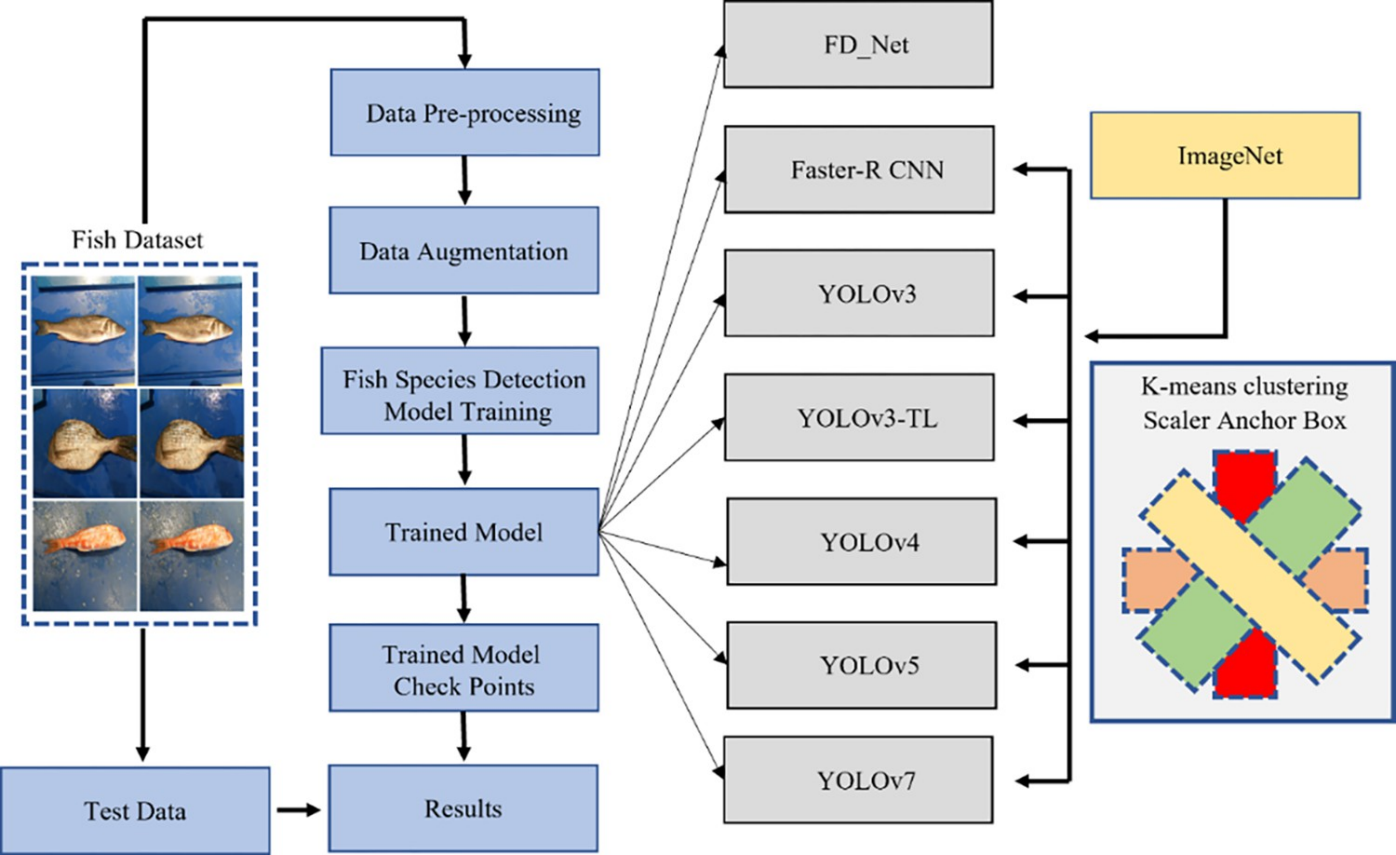
Study flow diagram for detecting several fish species.

This section consists of an experimental process that was carried out to measure the classification accuracy of the suggested model known as FD_Net, which is built on improved YOLOv7 with BNAM for the detection of nine different species of fish. This system was trained and validated using a dataset that was made accessible to the public and was described in the study [43]. The collected images have been scaled to the fixed size of resolution of the image i.e., 299 x 299 x 3. To prevent the model from being overly specific to the data, the process of data normalization was applied to the dataset. The dataset was cut up into three sections, which were designated as training, validation, and testing respectively. The experimental process was executed for up to 150 epochs. The performance of the FD_Net was examined and compared with YOLOv3 [77], YOLOv3-TL & YOLOv3-BL [78], YOLOv4 [79], YOLOv5 [80], Faster-RCNN [81], and the most recent YOLOv7 [82] in terms of the Intersection over Union (IoU), mean average precision (mAP), accuracy, sensitivity, precision, and the f1-score. The schematic block diagram of this study is shown in Fig 1.

### Dataset description

**Fig 2:**
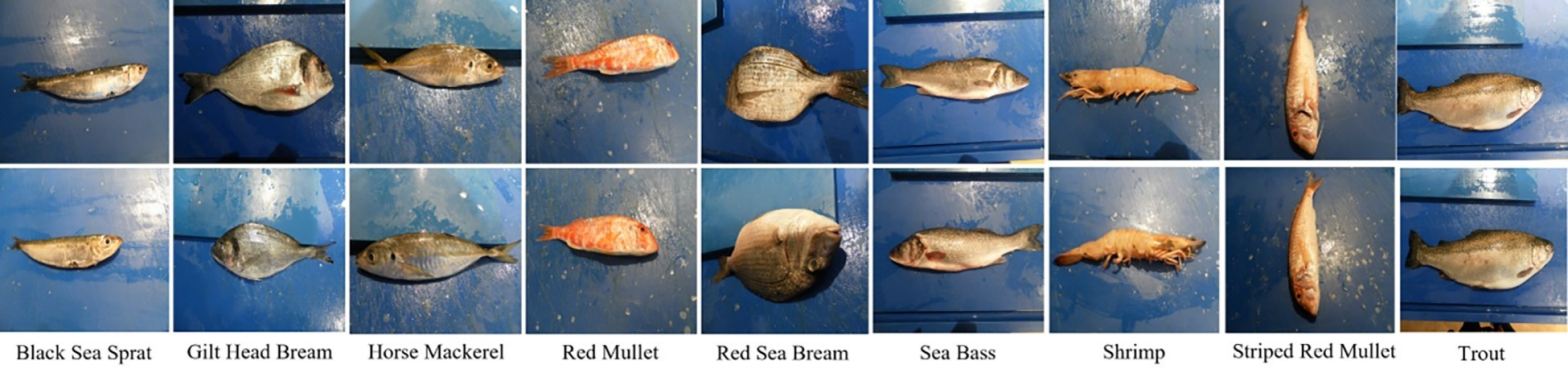
Sample fish images of the dataset.

This dataset [43] is available publically and contains images of nine distinct types of seafood procured from the fish counter of a grocery store. A Kodak Easyshare Z650 and a Samsung ST60 are the two cameras that are utilized during the process of gathering the information for this dataset. Although 50 unique fish photos are obtained for each of the nine classes such as gilt-head bream (GHB), red sea bream (RSB), sea bass (SB), red mullet (RM), horse mackerel (HM), black sea sprat (BSS), striped red mullet (SRM), trout (TRO), shrimp (SHR). Fresh fish is used in the process of acquiring the photograph, and while they are positioned in a variety of orientations and displacements, the lighting conditions do not dramatically shift throughout the procedure. Last but not least, to make the dataset useable in research that deals with real-life situations, a blue and noisy background is preferable over a spotlessly white background. Fig 2 is an illustration of several example photographs taken from the dataset that was collected.

In addition, the test images for all nine classes have had their dimensions reduced to the fixed size of resolution (299 x 299 x 3) while maintaining almost the same aspect ratio. The size of the dataset was increased through the application of the affine transformation [82]. Table 1 contains a comprehensive display of the dataset’s statistical information after synthetically increasing the size of the dataset.

**Table 1:** Summary of the fish detection dataset.

Sr#	Fish Type	Abbreviations	No. of images	Augmented Image
1	Gilt-head bream	GHB	50	1000
2	Red sea bream	RSB	50	1000
3	Sea bass	SB	50	1000
4	Red mullet	RM	50	1000
5	Horse mackerel	HM	50	1000
6	Black sea sprat	BSS	50	1000
7	Striped red mullet	SRM	50	1000
8	Trout	TRO	30	1000
9	Shrimp	SHR	30	1000
**Total**			**410**	**9000**

### Marking the bounding box of the fish dataset

The quality of fish objects varies from image to image depending on the direction in which the fish are swimming [83]. Therefore, determining which fish object should be given the best label is an interesting challenge. The dataset was broken up into nine different categories. Each class’s stored work was placed in the appropriate folder. In addition, the names of the images were changed to conform to the specifications, and each class of the dataset was given a name consisting of the respective class name, followed by GHB, RSB, SB, SRM, RM, HM, BSS, TRO, and SHR. Finally, the file that was produced after labeling the image had the extension.xml. The picture bounding box coordinates and the name of the class are automatically saved in the.xml file. Table 2 presents the bounding box coordinates to the dataset.

**Table 2:** Coordinate the format of the fish detection dataset for the bounding box.

Fish Type	Class	Xmin	Ymin	Xmax	Ymax
GHB	1	312	298	409	395
RSB	2	335	298	415	399
SB	3	331	295	413	255
RM	4	330	285	400	275
HM	5	306	299	455	315
BSS	6	365	300	399	299
SRM	7	312	265	369	258
TRO	8	417	285	369	301
SHR	9	396	258	411	322

### Proposed model

Fig 1 depicts the model structure of the proposed method for the identification of fish that live underwater. The improved YOLOv7 algorithm is used in the object detection module (ODM), which results in improved detection capabilities. The FD_Net method, which is described in this article, is implemented in the recognition module. This method makes use of a network for feature extraction and loss calculation. The improved DenseNet-169 is the network that is used for feature extraction, while the Arcface loss (AFL) function is used for the loss function.

#### YOLOv7 and ODM

In this particular investigation, the YOLOv7 [23] method is used for fish object identification. This decision was taken after some time was spent previously weighing the benefits of several YOLO series algorithms. To identify fish targets, we implemented several modifications to YOLOv7, one of which was the complete replacement of the feature extraction network as well as an upgrade to the existing feature extraction network [80]. These changes were done to classify nine different types of fish species. YOLOv7 is comprised of three fundamental components: the Backbone network, which is in charge of feature extraction; the improved feature extraction networks; and the YoloHead network, which is in charge of prediction [81]. The original YOLO network has been improved with the introduction of the YOLOv7 platform. There have been enhancements made to several different aspects, such as the feature extraction network, the activation function, the loss function, and several other areas. Both the activation function, which is changed from Leaky ReLU to Mish, and the network for feature extraction, which is changed from YOLOv7’s [82] Darknet53 to CSP Darknet53, are updated to reduce the size of the model while maintaining the same level of accuracy. The version of the YOLO algorithm known as YOLOv7 is both more accurate and more productive when compared to previous iterations of the YOLO algorithm [83].

#### Improvements to YOLOv7

MobileNetv3 has been used instead of the feature extraction network CSP Darknet53 in the YOLOv7 network to make the OBM more lightweight and user-friendly across mobile platforms [74]. According to [77], MobileNetv3 has the potential to be used for categorization and has a positive impact on feature extraction. [28] This is accomplished via the integration of the Squeeze and Excitation (SE) Network as well as by the modification of the complex tail structure of MobileNetv2. The result is a network that is more accurate and requires less processing power than MobileNetv2 [84]. If you choose MobileNetv3 as the backbone network for feature extraction for YOLOv7, you may achieve good detection results with fewer parameters; in addition, MobileNetv3 is more lightweight than CSP Darknet53 [85].

Multiple researchers [47–58] have looked at the possibility of using MobileNet in conjunction with YOLO as a method. The authors of the research [29] concluded that an upgraded version of MobileNetv3 would be a better choice than Darknet53 in YOLOv3 for feature extraction since it would both lower the overall complexity of the algorithm and make the model more accurate [86]. In addition, channel attention and spatial attention in MobileNetv3 come together to build a whole new attention module known as SESAM. In the article [85], the authors presented a structure that was based on the YOLOV5-MobileNetv3Smal network model. They also applied MobileNetv3Smal to YOLOv5, which improved the Backbone network structure by resolving the problem of inference of high-pixel pictures using excessive memory on low-power edge computing nodes. According to the research [32], the backbone of YOLOv4 is built on MobileNetv3, which is improved by CBAM and adapted from SENet. As a consequence of this, the complexity of the model is reduced, and the effect of interference from high-light backgrounds is reduced to a minimum [87].

**Fig 3:**
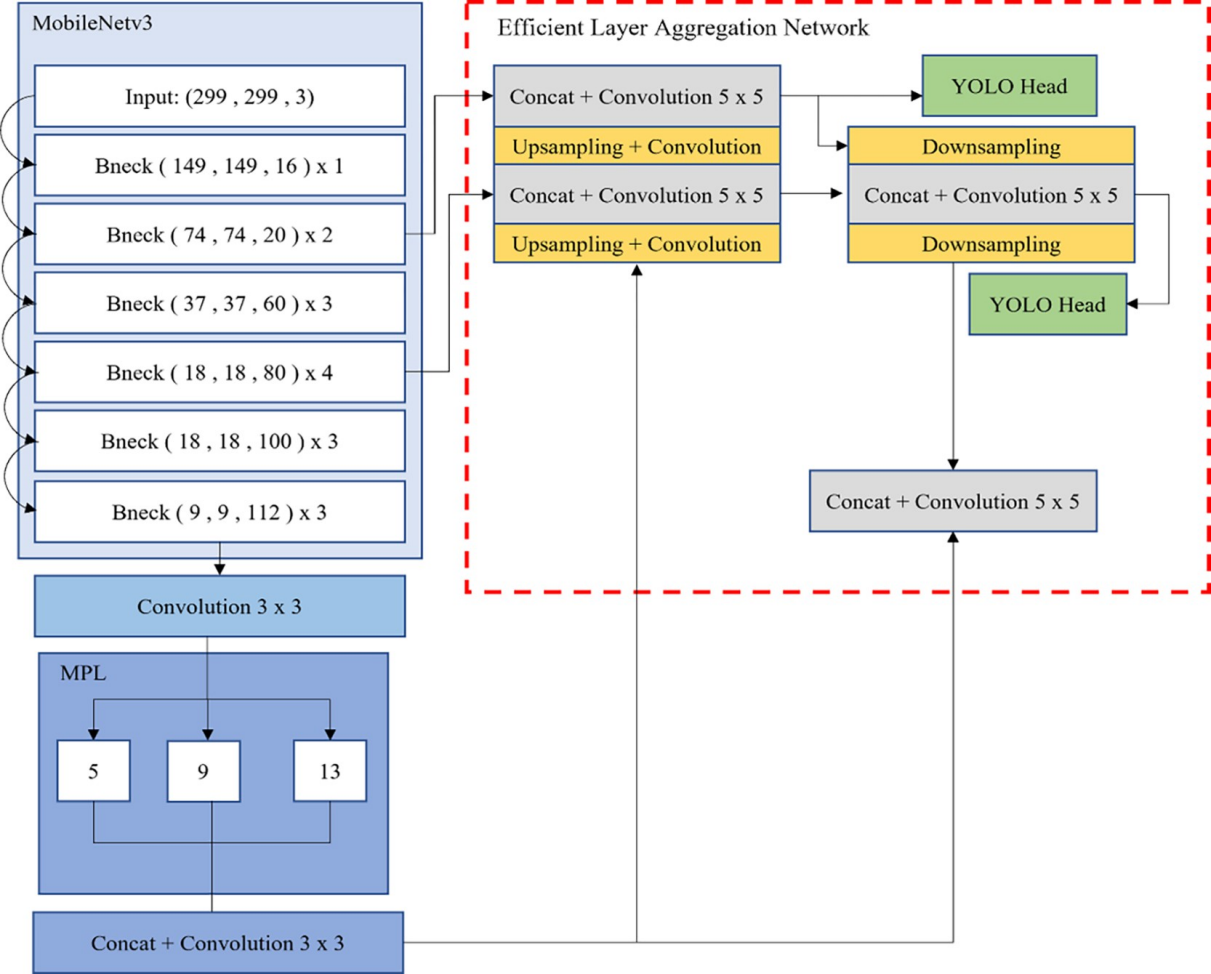
Enhanced YOLOv7 architecture.

The problem of fish species identification was addressed by this work by modifying CSP Darknet53 in YOLOv7 to MobileNetv3 [88]. Additionally, the input feature size was increased to 299 x 299, and the output channels of the three effective feature layers were set to 20, 60, and 80, respectively. YoloHead is then connected to the three effective feature layers that were previously created. Additionally, an application of depthwise separable convolution [85] is investigated in this study for YOLOv7. By using depthwise separable convolution, one may split the spatial dimension from the channel dimension during the convolution process [89]. The input and output are the same as in a standard convolution, but it uses a much smaller number of parameters and calculations. As a result, including it in the network might result in a considerable reduction in the total number of parameters as well as the amount of time required for computation. Some researchers have also looked at whether or not depthwise separable convolution applies to certain situations. In the feature extraction network described in [36], the regular convolution is switched out for the depthwise separable convolution. In addition, the attention mechanism is added in the channel and spatial dimensions of each dense block of the feature extraction network to focus on tiny targets. In the study [37], the conventional dense bottleneck block is changed into a compact dense bottleneck block by removing the very last 1x1 convolution layer and replacing it with a 3x3 depthwise separable convolution. This research makes use of depthwise separable convolution on the enhanced feature extraction network i.e., Extended Efficient Layer Aggregation Network (E-ELANet) [90,91]. Additionally, all three convolutions in E-ELANet are replaced with depthwise separable convolutions to further reduce the number of parameters and speed up the calculation. Fig 3 shows the improved structure of YOLOv7.

#### FD_Net model

With the fish identification module, this body of work presents a method known as FD_Net, which is an underwater fish recognition methodology. It is made up of two parts, namely, the loss computation and the backbone feature extraction network. The loss function is modified to Arcface Loss (AFL), and the backbone network that is used for feature extraction is improved based on DenseNet-169 [92]. The following is a list of the most important improvements made to DenseNet-169:

1. The BNAM is integrated inside the dense block of DenseNet-169.
2. Before the convolution process begins, the dense block goes through batch normalization (BAN).
3. While the max-pooling layer (MPL) is eliminated and dilated convolution is added to the dense block, the conventional convolution is maintained as the primary method of processing data in the backbone network.
4. Instead of the ReLU activation function, we employ the Hard-Swish activation function
5. BAN–dropout–Fully Connected (FC)–BAN strategy is used in this work.

**Fig 4:**
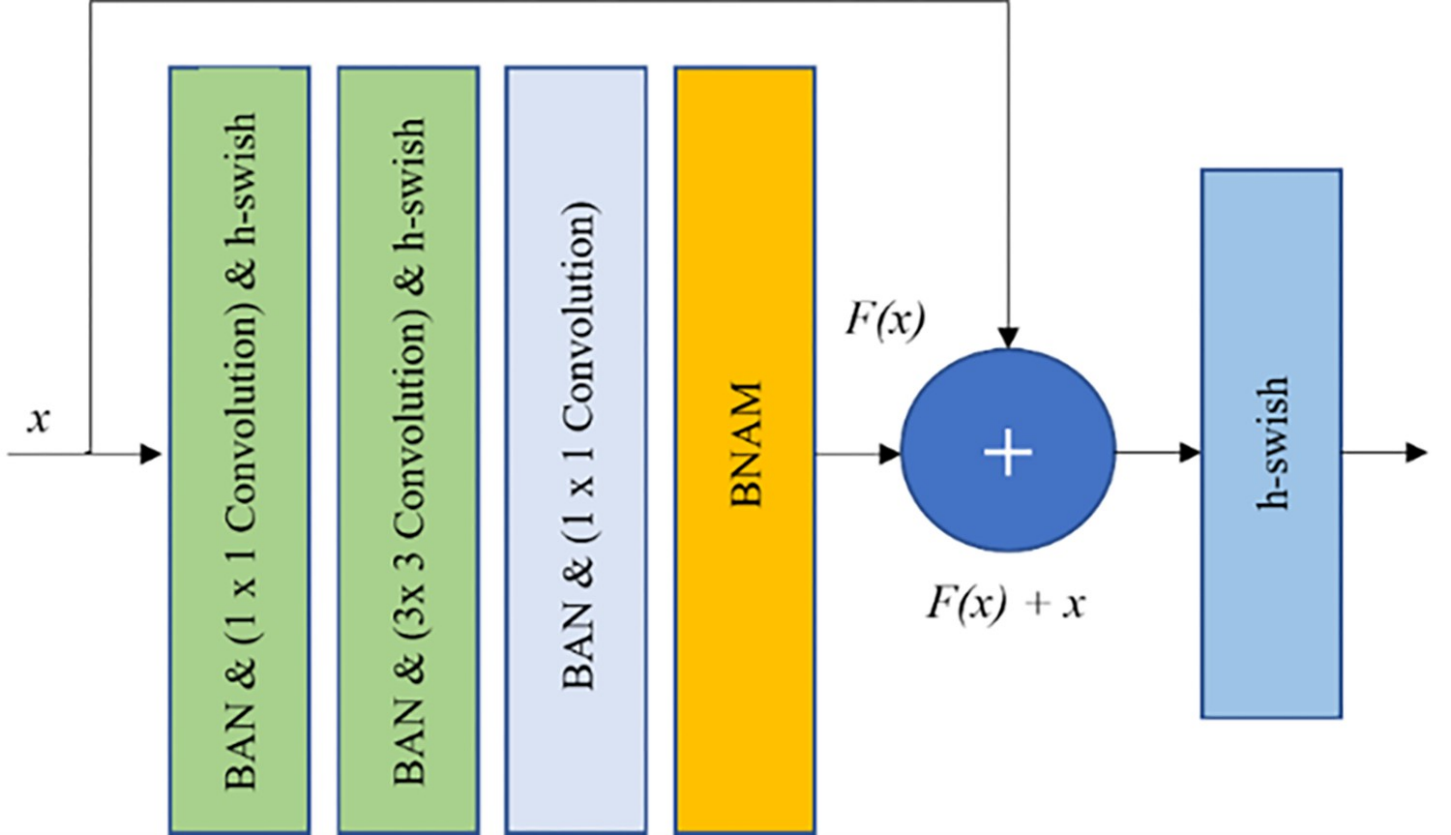
P-Bottleneck: Dense block after embedding BNAM.

**Fig 5:**
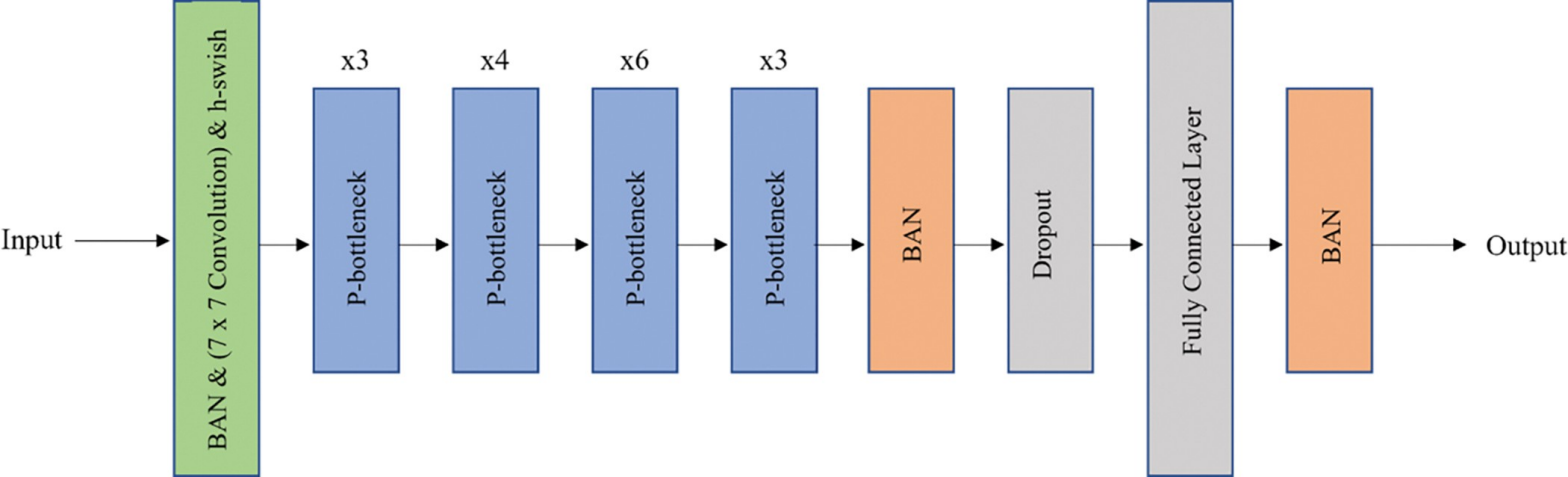
Backbone network DenseNet-169.

Figs 4 and 5 illustrate, respectively, the structure of the dense block of the enhanced DenseNet-169, which has been given the designation P-Bottleneck, and the structure of the improved backbone network.

#### DenseNet-169

**Fig 6:**
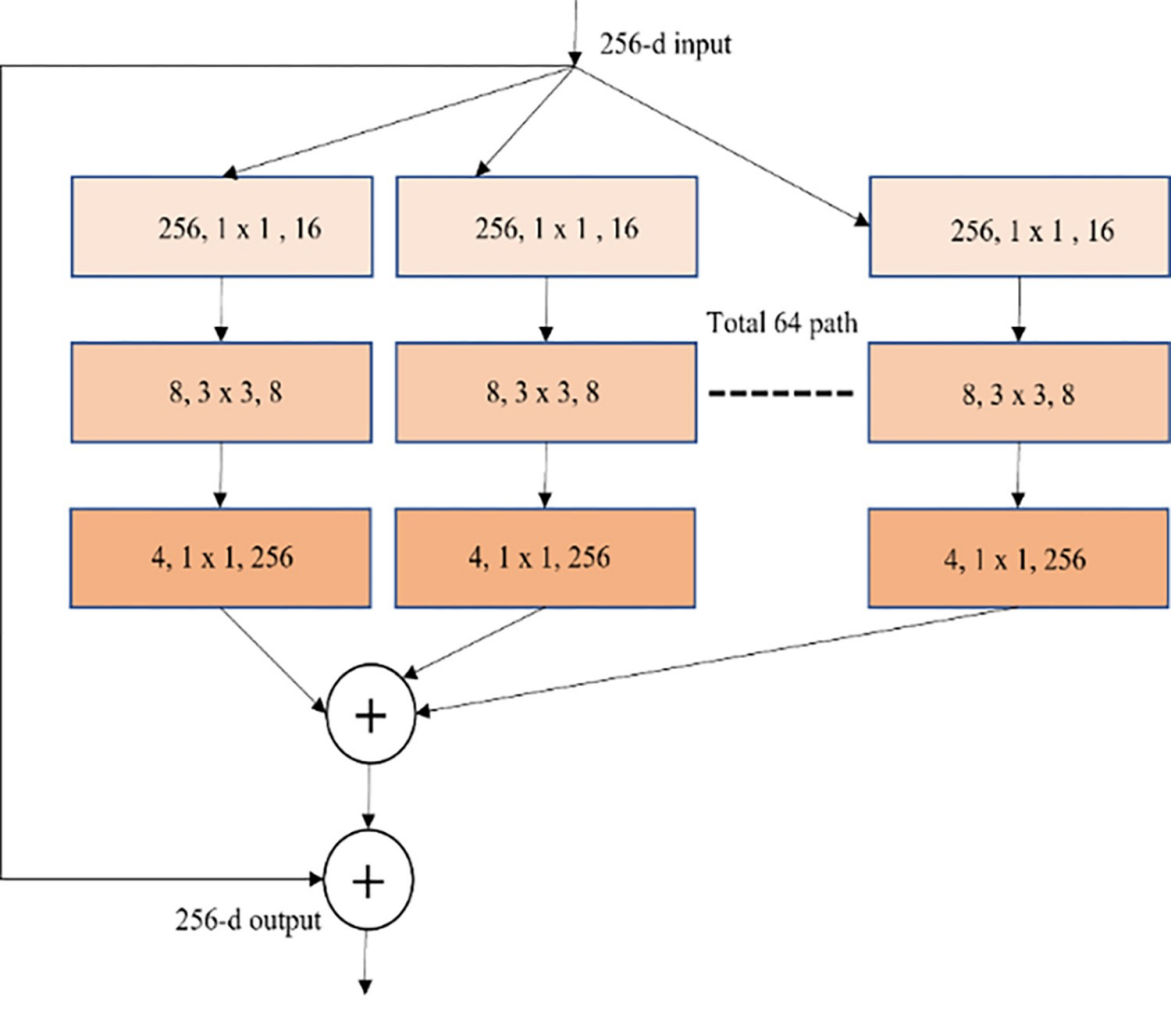
The block structure of DenseNet-169.

This research utilizes and improves the DenseNet-169 network because, in comparison to DenseNet-121 and ResNet, the performance of DenseNet-169 is much higher [90–93]. Cardinality is a new hyperparameter that has been added to DenseNet with version 169 [92]. It was stated in the cited work [93] that increasing cardinality is a more effective strategy for attaining accuracy than expanding either the depth or the breadth of the considerations [94]. The input channels are split up into a greater number of groups for the convolution process when the cardinality is increased. This results in the output channels being wider and having a greater number of features. The DenseNet-169 block structure is seen in Fig 6.

**Fig 7:**
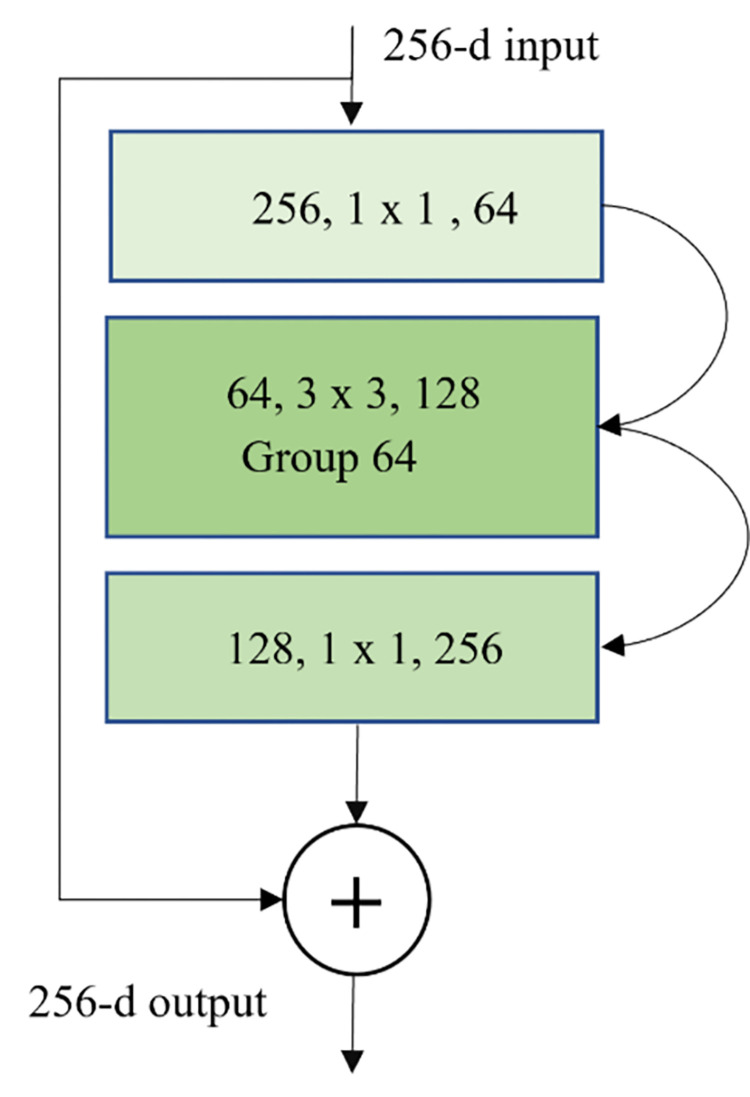
Unit of DenseNet-169.

As can be seen in Fig 6, the input of the DenseNet-169 block is a 256-channel feature map. This map is then divided into 64 branches, each of which has 256 input channels for the first convolutional layer, a 1x1 convolution kernel, and 8 output channels [95]. The number of input channels in the second convolutional layer of each branch is set to four, the kernel size is three, and the number of output channels is likewise set to four. The number of input channels in the third convolutional layer of each branch is set to 4, the kernel size is set to 1, and the number of output channels is set to 256 [96]. After that, separate additions are made to each of the output feature maps of the 64 branches. The final output is generated by adding the result of the summation to the input piece, which is done by a rather straightforward connection. The structure that may be created by simplifying Fig 6, which is the structure that is used the most often, can be seen in Fig 7.

#### Bottleneck attention module (BNAM)

**Fig 8:**
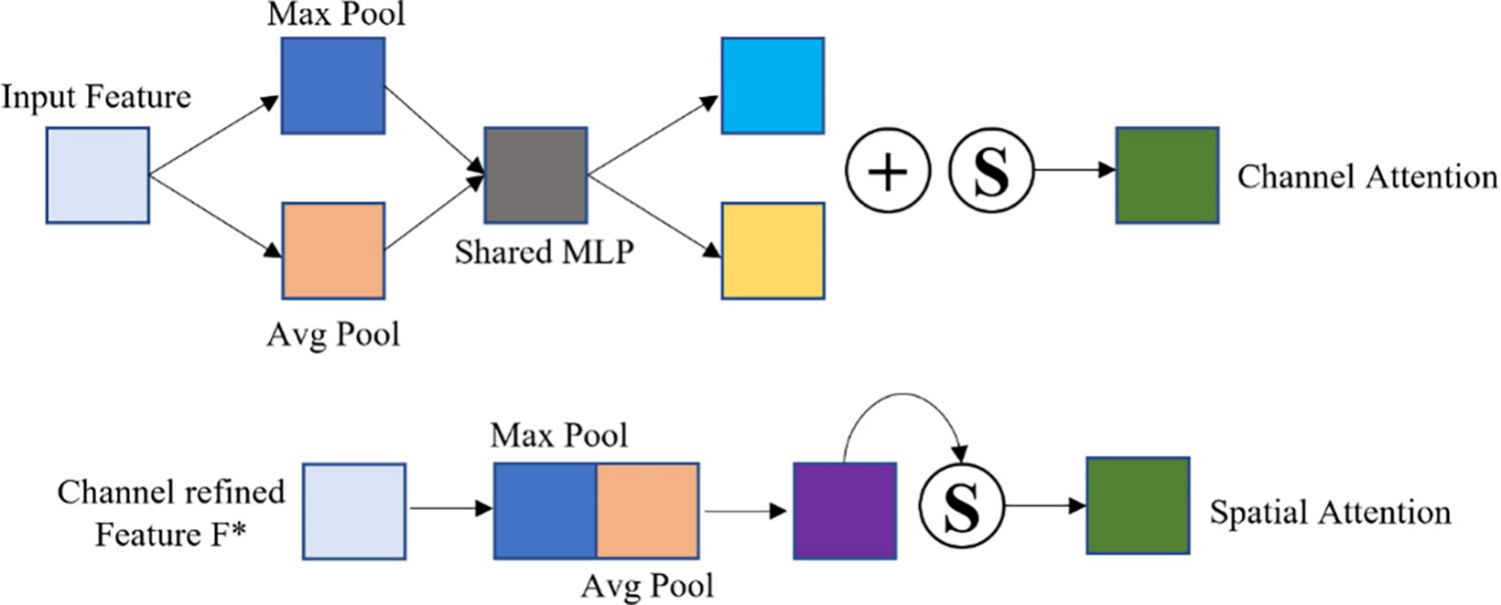
The structure of BNAM contains two modules i.e., channel and spatial attention.

The purpose of incorporating the BNAM into the dense blocks of DenseNet-169 is to enhance the capability of the network to extract properties shared by a variety of fish species. A lightweight attention module that is introduced by [90], it is made up of the Channel Attention Module (CHAM) and the Spatial Attention Module (STAM) as depicted in Fig 8.

The input feature maps are initially pooled in the CHAM by using maximum and average pooling, in that order, before being sent to the Shared MLP for processing. The Shared MLP is then followed by the inclusion of the individual output feature components to integrate the output features. Activating the sigmoid function is the last step in the process of extracting the output characteristics of the CHAM. The characteristics that are generated by the CHAM are taken as input by the STAM, which then makes findings based on those characteristics. In addition, maximum pooling and average pooling are carried out before splicing the two layers together and carrying out the convolution procedure to reduce the channel to a value of 1. In the end, sigmoid activation is a method that may be used to get the output qualities of the STAM. In [97], the BNAM attention module is applied to the output of DenseNet-169 to perform BNAM processing on each group of the detailed features to get useful features, suppress superfluous features, and enhance information usage. We used the BNAM on the dense block of DenseNet-169 for the fish species identification challenges. After embedding the BNAM, we renamed the dense block as B-Bottleneck and subsequently replaced Bottleneck with P-Bottleneck. The purpose of this is to improve the network’s ability to extract the characteristics of each fish and extract features with a higher level of detail.

#### Batch Normalization (BAN)

The input data are converted into a normal distribution via BAN [92], which has a mean value of 0 and a variance value of 1. Using this approach, the network’s capacity for generalization, initial learning rate, and convergence speed may all be improved. In addition, the initial learning rate of the network can also be boosted. First, the convolution operation is carried out on the dense blocks of the initial DenseNet-169 network. Next, the effective BAN operation is carried out on these blocks. However, when training is being done, it is possible to make the network unstable, and it is also simple for the loss to drop to cause enormous fluctuations; both of these things would reduce the overall influence that the training has. To make the training process more reliable and to make it go even more quickly, this research proposes moving the BAN layer up in the hierarchy and putting it in front of the convolutional layer. The data that is being input from a higher layer is first put through a BAN operation to ensure that it follows the normal distribution, and then it is put through a convolution operation, which may increase training speed and make the training process more stable. The data that is being brought down from a higher tier is being processed by both of these actions as it comes in.

#### Pooling layers

When DenseNet-169 was originally implemented, the input feature map was subjected to maximum and average pooling. When performing the downsampling operation on the feature map, the highest value of the neighborhood’s feature values is used as the starting point for the procedure [98]. The size of the feature map is reduced by half after the max-pooling layer, which may function as dimension reduction and lower network parameters. However, in the process of dimension reduction, some details and more manageable objectives will be lost. Since the information that is lost cannot be recovered, the end product will not be perfect. The goal of average pooling is to determine the value of the neighborhood’s feature values on average. This may be able to effectively maintain the background, but it also can rapidly blur the picture. Given that the visuals that correlate to the various perceptual fields of the various points are unique from one another, the relative weights that are assigned to the various points need to also be distinct from one another. However, the performance of the network is degraded when they are handled in the same manner as if they had the same weight, which is how the average pooling method works.

Because of the difficulties caused by the layer that is directly above it, the pooling layer of the DenseNet-160 backbone network has been modified in this research. First, max pooling will no longer be used to maintain the same size of the feature map and prevent the loss of a significant quantity of data. After that, the layer responsible for averaging the pooled data is removed, and the BAN–dropped–FC–BAN structure is put into place. When the average pooling is taken out of the equation, the information from the original feature map may be kept without any image distortion taking place. At the moment, connecting a BAN layer to the network may normalize the input data. This is then followed by a dropout layer, which prevents overfitting by randomly deactivating neurons in the network. After that, it moves on to the fully linked layer, where it begins to extract properties that enable categorization. The very last layer is a BAN layer that uniformly regularizes the output data to deliver the very last output features.

Since the max-pooling layer can cause the image’s receptive field to increase, the original picture’s receptive field, which corresponds to the created feature map, would shrink if max pooling is immediately disabled. The subsequent convolution operations will be impacted as a result of this circumstance. To improve the receptive field, the authors of this research implement dilated convolution inside a succession of dense structures of DenseNet-169. However, the backbone network, which does not include the dense structure, continues to make use of standard convolution.

#### Dilated convolution and activation function

Dilated convolution [99] is an extension of standard convolution that involves the injection of holes to increase the size of the receptive field while preserving the dimensions of the initial input feature map. Dilation convolution is an extension of traditional convolution that involves the addition of a new hyperparameter known as the dilation rate. This hyperparameter allows the user to choose the number of spaced kernels. In this investigation, we make use of the Hybrid Dilated Convolution (HDC) [90] algorithm that is described in the study [94], and we apply three different expansion rates 1, 2, and 3 for each convolutional layer. In this way, the information contained inside each pixel will be preserved, and the receptive field will be enlarged.

In the first implementation of DenseNet-169, the ReLU activation function was put to use. Although the ReLU activation function is used rather often, there are a few restrictions on its use. When the input is either close to zero or negative, the ReLU function gradient will eventually hit zero. When this occurs, the network will be unable to finish backpropagation, which will result in neuron deactivation. To avoid these kinds of problems, this research makes use of the Hard-Swish (H-Swish) activation mechanism [100].

The H-Swish activation function was suggested by Prajit Ramachandran et al [97]. This activation function is an improvement over the Sigmoid and ReLU activation functions. Swish combines the advantageous aspects of the Sigmoid and ReLU activation functions, and as a result, the performance of its deep model is superior to that of both. The expression of the sigmoid and H-Swish function is mentioned in Eqs (1) and (2) respectively:

F(S)=S×Sigmoid(L×S) (1)

$$H-Swish(h)=\{\begin{array}{cc} 0, & h\leq -3\\ h & h\geq 3\\ h\times \frac{\left(h+3\right)}{6}, & otherwise \end{array}$$ (2)

where L represents the learnable parameter. Through the use of H-Swish, this research was able to effectively replace all of the ReLU activation functions that were included in the initial DenseNet-169 network.

#### Loss function

The four coordinates that are predicted by the FD_Net, YOLOv3, YOLOv3-TL, YOLOv3-BL, YOLOv4, YOLOv5, Faster-RCNN, and YOLOv7 model architecture for each bounding box are tX, tY, tW, and tH. The Intersection over Union (IoU) [82] metric is the sort of evaluation metric that is utilized for object recognition more frequently than any other type. The degree to which the anticipated bounding box overlaps with the ground truth is displayed by this statistic. The coordinates of the upper left corner are commonly used to represent the ground truth, whereas the coordinates of the bottom right corner are commonly used to represent the forecast box (x_1_, y_1_, x_2_, y_2_). Because this is a vector, however, its distance can normally be calculated by applying the L_1_ and L_2_ norms to the vector. Even though the L_1_ and L_2_ norms both assume the same value, the actual detection impact of IoU and generalized intersection over union (GIoU) is drastically different [86]. If the projected bounding box does not coincide with the ground truth, then the IoU values will be deemed to be zero in that situation. The fact that the gradient of the outcome is 0 demonstrates that optimization cannot be performed successfully. On the other hand, IoU offers a considerable issue whenever it appears that the predicted box and the ground truth do not overlap. It does not reflect the distance that is necessary to separate the ground truth from the forecast box in an appropriate manner [101]. To work around this limitation of IoU, this study suggests using GIoU as a new bounding box loss function instead of using it. The coordinate regression loss by GIoU is represented by Eqs (3 & 4), which can be calculated as follows:

$$IoU=\frac{\left|A\cap B\right|}{\left|A\cup B\right|}$$ (3)

$$GIoU=IoU-\frac{\left|C\backslash (A\cap B)\right|}{\left|C\right|}$$ (4)

The parameters *A* and *B* stand for the expected truth and the ground truth, respectively, in each of the boxes. The parameter *C* represents the smallest convex region that includes both *A* and *B* in its scope of coverage. When the expected and ground truth bounding boxes coincide, Eqs (3&4) show that the values of both IoU and GIoU are reasonably close to value 1, indicating that this occurs when the two sets of bounding boxes coincide. If there is no overlap between the values that are expected and the values that are found on the ground, then the IoU will be equal to zero, and the GIoU will finally be equal to one. As a consequence of this, we concluded that we should refer to this function as 1 –GIoU loss [92]. When there is a greater distance between the bounding boxes that are predicted and those that represent the ground truth, the ranges of IoU and GIoU are [0, 1] and [–1, 1], respectively. The bounding box regression loss (BBRL) [93] of GIoU is characterized by Eq (5).


BBRL=1−GIoU (5)

The proposed FD_Net model confidence loss and classification are calculated by using Eqs (6) and (7):

$$C_{loss}=\sum_{X=0}^{A_{2}}*\sum_{Y=0}^{B}l_{XY}^{object}(I_{x}-\bar{I_{x}}{)}^{2}+{⋋}_{XY}\sum_{X=0}^{A_{2}}*\sum_{Y=0}^{B}l_{XY}^{No-object}(I_{x}-\bar{I_{x}}{)}^{2}$$ (6)

$${Class}_{loss}=\sum_{X=0}^{A_{2}}*l_{XY}^{object}*\sum_{classes}^{B}\left(H_{i}(C)-J_{i}(C\right){)}^{2}$$ (7)

The confidence loss is defined by Eq (6), where $l_{XY}^{object}$ is responsible for the existence of the target object in a grid *I* by the *Y*_*th*_ bounding box, *A*^*2*^ represents the grid cells, and *B* represents the bounding box. *I*_*x*_ is the actual data of the box confidence score, and $\bar{I_{x}}$ is the box confidence prediction score. Class loss is defined by Eq (7), where $l_{XY}^{object}$ is accountable for whether or not an object exists in cell *C*, *H*_*i*_ *(C)* is the real object probability confidence score, and *J*_*i*_ *(C)* is the forecast value.

The loss function is very necessary for ensuring that the model training effect is achieved and that correct prediction are made. Some of the most common types of loss functions are the Softmax Loss [98], the Triplet Loss [99], and the Arcface Loss [100]. An illustration of the Softmax Loss equation may be found in Eq (8). Because it does not need intra-class compactness, it is not suited for individual recognition tasks. However, it may guarantee the separation of categories in fish identification.


$$Softmax(S)=-\frac{1}{N}\sum_{i=1}^{N}\log\frac{e^{w_{y}^{U}(x_{i}+by_{i})}}{\sum_{j=1}^{n}e^{W_{y}^{U}(j+by_{j})}}$$ (8)

After some time, a large number of researchers developed variants of the Softmax Loss algorithm to enhance its capacity for discrimination. The Arcface Loss was discussed in [101], and it was based on the formula for the Softmax Loss (see Eq (9)).


$$Arcface(A)=-\frac{1}{N}\sum_{i=1}^{N}\log\frac{e^{s(\cos\left(\theta_{y_{i}}+m)\right)}}{e^{s(\cos\left(\theta_{y_{i}}+m)\right)}+\sum_{j=1,j\neq y_{i}}^{n}e^{s(\cos\left(\theta_{j})\right)}}$$ (9)

After training using Arcface Loss, it is feasible to obtain larger fish species class distances, constant performance without mixing with other loss functions, and simple convergence. As a result, the Arcface Loss was selected to serve as the loss function for the fish identification network in this particular paper.

## Results and discussions

For this study, a total of eight networks, including FD_Net, YOLOv3, YOLOv3-TL, YOLOv3-BL, YOLOv4, YOLOv5, Faster-RCNN, and YOLOv7 have been trained and tested, and the results of each network are provided here. The findings were drawn from a fish dataset that was one of a kind and consisted of nine different categories such as GHB, RSB, SB, SRM, RM, HM, BSS, TRO, and SHR.

### Experimental setup

The neural network models i.e., FD_Net, YOLOv3, YOLOv3-TL, YOLOv3-BL, YOLOv4, YOLOv5, Faster-RCNN, and YOLOv7 were constructed with the assistance of the computer vision libraries OpenCV [94] and Keras [86]. In addition, Python is used for the programming of various methods that are not immediately related to neural networks. The experiment was done on a computer running Windows, which had both a 32 GB graphics processing unit (GPU) and an 11 GB NVIDIA graphics processing unit installed.

### Performance evaluation

To determine the accuracy of the proposed FD_Net, YOLOv3, YOLOv3-TL, YOLOv3-BL, YOLOv4, YOLOv5, Faster-RCNN, and YOLOv7 models, the following methods were implemented: IoU, GIoU, mean average precision (mAP), and precision-recall. In addition, the detection time as well as the frame rate per second (FPS) [95] is a significant assessment index that can be used to measure the effectiveness and speed of the network when it is offline. In addition to this, it was found that the predicted value of the bounding box’s IoU was accurate, and a rise in the threshold value led to an increase in the number of overlaps in the ground truth. During the process of computing the association between the bounding box of the ground truth and the prediction, this measure played an important role. If the IoU value is higher than the threshold value, the detection result will be regarded as correct. For us to move on with this work, we will be utilizing GIoU as a method for determining the typical precision of our detection model. Our major goal is to calculate the mean average precision (mAP) [96] that occurs between the bounding boxes that were predicted and the ground truth. If the value of the IoU is higher than the threshold of 50%, the result of the test will be regarded as a true positive (TP) [98]. On the other hand, the result of the test will be considered a false positive (FP) if the IoU value of the model is greater than 50% of the threshold but our model considered this value lower than the threshold. The image does contain a second object, even though the false-negative (FN) [99] result shows that the image does not contain the object that was being looked for. If the value of the IoU is lower than the threshold of 50%, the result of the negative will be regarded as a true negative (TN). In this particular study, the measurements that were previously used to calculate recall and precision are combined. This was done so that the results may be more accurately interpreted. The average precision (AP), when extended, can be used to compute the mean absolute precision (mAP) of each class. For this thesis, there are a total of nine distinct species of fish will be utilized to compute the mean of average precision, and the mAP index will be the metric that will be employed to determine how accurate the results are. Precision (PRE), accuracy (ACU), f1-score, specificity (SPF), and recall (REC) [100] have each been calculated based on the number of true positives (TP), true negatives (TN), false positives (FP), and false negatives (FN), in that order. Eqs (10–14) are employed, to define the terms PRE, REC, ACU, SPF, and an F1-score, respectively.


$$PRE=\frac{TP}{TP+FP}$$ (10)

$$REC=\frac{TP}{FN+TP}$$ (11)

$$ACU=\frac{TP+TN}{TP+TN+FN+TP}$$ (12)

$$F1-score=2*(\frac{PRE*REC}{PRE+REC})$$ (13)

$$SPF=\frac{TN}{TN+FP}$$ (14)

Where TP is the detection of an object correctly with a positive sample, and FP is the detection of an object adversely by the error of a positive sample, TN indicates the total number of correctly labeled negative cases and FN value, which refers to the total number of positive samples that were incorrectly labeled as negative.

### Results analysis and discussions

**Fig 9:**
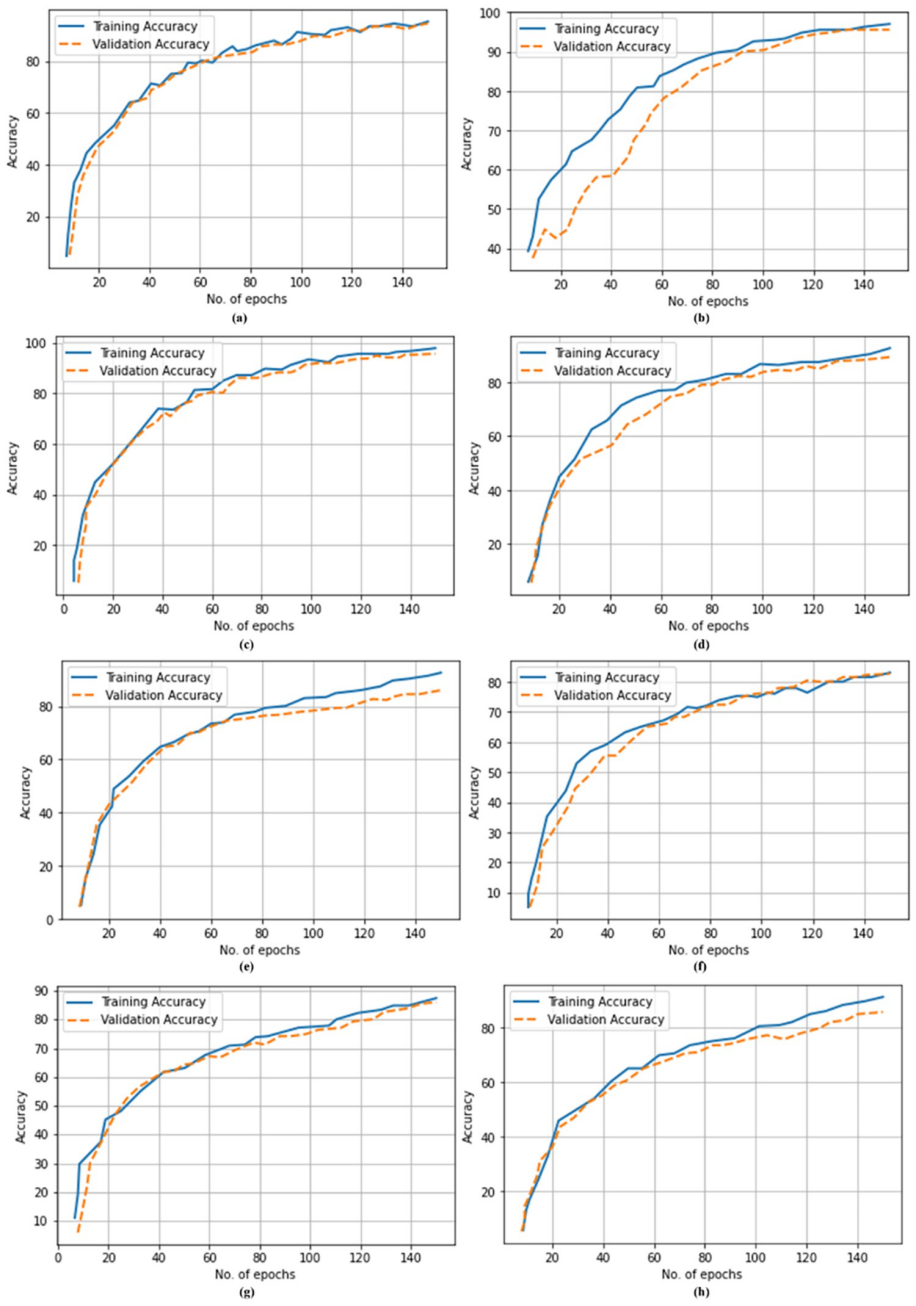
Training and validation accuracy curves of the a) FD_Net model, b) YOLOv3, c) YOLOv3-TL, d) YOLOv3-BL, e) YOLOv4, f) YOLOv5, g) Faster-RCNN, and h) YOLOv7.

**Fig 10:**
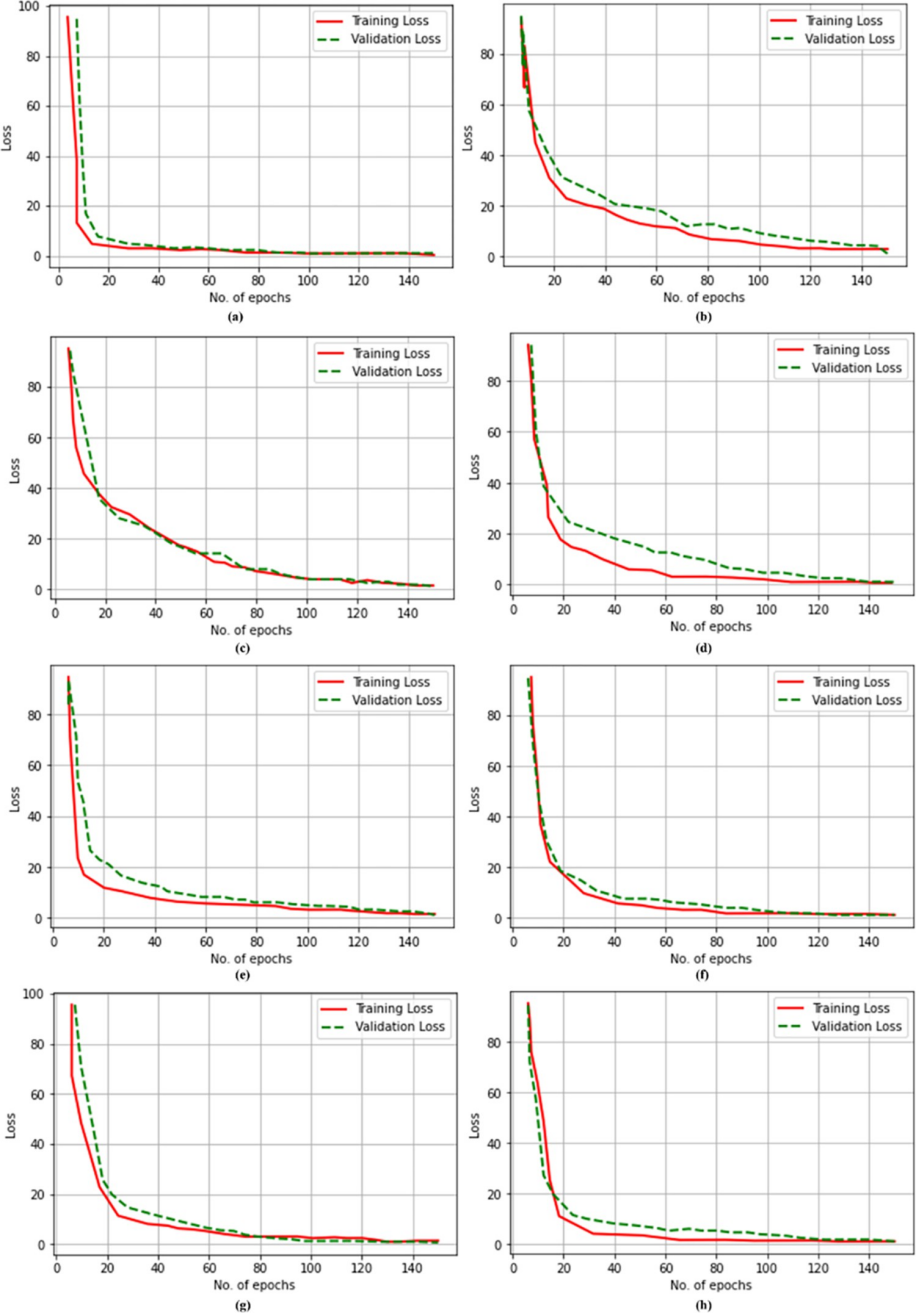
Training and validation loss curves of the a) FD_Net model, b) YOLOv3, c) YOLOv3-TL, d) YOLOv3-BL, e) YOLOv4, f) YOLOv5, g) Faster-RCNN, and h) YOLOv7.

The large fish dataset [43] is utilized in the process of fixing and initializing the darknet-53 backbone architecture that is used for fish detection tasks. When the model is being trained, it is necessary to make use of images that have a resolution that varies between scales. When there are 32 batches, the resolution of the dataset is applied to the fixed input image once every batch. For the FD_Net, YOLOv3, YOLOv3-TL, YOLOv3-BL, YOLOv4, YOLOv5, Faster-RCNN, and YOLOv7 we set the start and final learning rates at 0.05 and 0.01, respectively, during the training stage. The batch size is 32, the IoU threshold is 0.5, and the average decay is 0. The proposed FD_Net and other YOLO models are trained for 150 epochs. To avoid the model from failing to converge during training, the learning rate is adjusted. Fig 9 depicts the training and validation accuracy curves for the proposed FD_Net, YOLOv3, YOLOv3-TL, YOLOv3-BL, YOLOv4, YOLOv5, Faster-RCNN, and YOLOv7. Additionally, Fig 10 represents the training and validation loss of these eight models.

#### Checkpoint size and parameters

All of the weight, gradients, biases, and other variable values are saved in a binary file with the extension ckpt, which is the checkpoint [96]. This checkpoint binary file is where TensorFlow [97] stores all of the variables that are used to convert variable names to tensor values. Additionally, it analyses each value on its own to determine the optimal mAP for storing the checkpoints (CP) files on the disc in a consistent manner. During the training, the checkpoint file was saved every five minutes as part of this study. The combined storage capacity of all checkpoints across all eight networks amounts to 52GB. The storage capacity of a single checkpoint size and the number of parameters is outlined in Table 3. It demonstrates that FD_Net is significantly less cumbersome and quicker than YOLOv3, YOLOv3-TL, YOLOv3-BL, YOLOv4, YOLOv5, Faster-RCNN, and YOLOv7, in addition to having fewer parameters.

**Table 3:** Specifications and sizes of the single checkpoint files for FD_Net, YOLOv3, YOLOv3-TL, YOLOv3-BL, YOLOv4, YOLOv5, Faster-RCNN, and YOLOv7.

Models	Single File CP Size	Parameters
FD_Net	115.6 MB	32 million
YOLOv3	585.1MB	45 million
YOLOv3-TL	585.1MB	45 million
YOLOv3-BL	799.3 MB	67 million
YOLOv4	985.2 MB	72 million
YOLOv5	675.6 MB	58 million
YOLOv7	697.5 MB	60 million
Faster-RCNN	756.6 MB	63 million

#### mAP of fish dataset

GHB, RSB, SB, SRM, RM, HM, BSS, TRO, and SHR. were nine types of fish that were included in a large number of fish species detection datasets [43] that were used in the experiment for this study. The total number of images for training is 6300 and 1800 images for validation, while there are 900 images for the test. When compared to the mAP of the YOLOv3, YOLOv3-TL, YOLOv3-BL, YOLOv4, YOLOv5, Faster-RCNN, and YOLOv7 model, which was trained from the ground up, the mAP of the FD_Net model achieves superior results with 150 training epochs. In this study, depthwise separable convolution was utilized to accomplish the goal of creating a lightweight network. According to the findings, FD_Net is capable of maintaining a high detection speed despite a moderate reduction in map size. The mAP comparison on the fish testing dataset is displayed in Table 4 at a 299 x 299 x 3 resolution for each algorithm. When contrasted with the YOLOv3, YOLOv3-TL, YOLOv3-BL, YOLOv4, YOLOv5, Faster-RCNN, and YOLOv7 models, the FD_Net model’s mAP performance is superior. When compared to other target identification algorithms, the mAP displayed unusually high levels of stability and discrimination [102]. In addition to this, it offers a single-figure evaluation of quality in comparison to memory levels. During the experiment that was conducted for the present work, the number of training epochs ranged from 0 to 150. The detailed summary of mAP results obtained for the eight YOLO models is presented in Table 4.

**Table 4:** Comparison of proposed FD_Net and seven YOLO models using the fish dataset at 299 x 299 x 3 image resolution.

Sr#	Models	mAP	PRE	REC	F1-Score	SPF	ACU
1	FD_Net	95.30%	95.15%	95.45%	95.39%	95.28%	95.29%
2	YOLOv3	87.69%	86.99%	87.12%	87.23%	87.03%	87.68%
3	YOLOv3-TL	88.52%	88.50%	88.45%	88.19%	88.19%	88.50%
4	YOLOv3-BL	87.93%	87.90%	87.96%	87.91%	87.26%	87.90%
5	YOLOv4	88.88%	88.88%	88.89%	88.59%	88.62%	88.86%
6	YOLOv5	87.93%	87.95%	87.92%	87.59%	87.79%	87.90%
7	Faster-RCNN	85.79%	85.80%	85.81%	85.78%	85.79%	85.76%
8	YOLOv7	90.49%	90.99%	90.25%	90.95%	90.91%	90.42%

**Fig 11:**
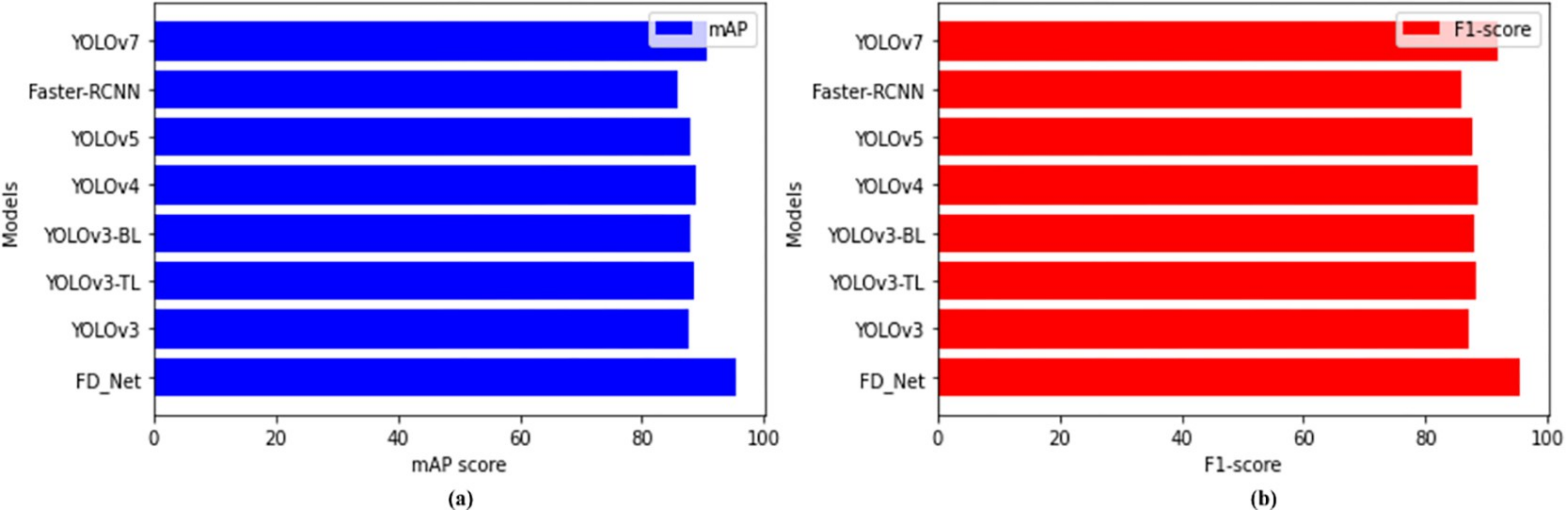
The mAP and F1-score are achieved by the proposed FD_Net and seven different models.

It has been observed that (see Table 4), the proposed FD_Net model achieved the highest mAP value (95.30%), F1-score (95.39%), SPF (95.28%), PRE (95.15%), REC (95.45%), and ACU (95.29%). The YOLOv3 achieved the 87.69% of mAP. The YOLOv4, YOLOv5, and Faster-RCNN achieved the mAP of 88.88%, 87.93%, and 87.93%, respectively. The YOLOv7 achieved an mAP of 90.49%. Fig 11 shows the graphical representation of the mAP and F1-score achieved by the proposed FD_Net and seven different models.

#### Analyzing mAP value by using different sizes of fish image resolution

As discussed earlier, one of the most important metrics to look at when evaluating the performance of a network on a testing dataset is the precision-recall ratio. This ratio compares the number of correct predictions to the total number of possible correct predictions. In addition, a measure that is taken for accuracy is the relevancy of the results, and a measure that is taken for the recall is the total number of correct results and relevant results. In this work, we additionally analyze the detection performance of the model by calculating the average percentage of false positives for each class and architecture using images of various sizes. The detailed mAP results achieved by the proposed model and other YOLO versions with different image resolutions are presented in Table 5.

**Table 5:** Comparison of proposed FD_Net and seven YOLO models in obtaining mAP value by using the fish dataset at various resolutions.

Image Resolutions	FD_Net	YOLOv3	YOLOv3-TL	YOLOv3-BL	YOLOv4	YOLOv5	Faster-RCNN	YOLOv7
299 x 299 x 3	95.30%	87.69%	88.52%	87.93%	88.88%	87.93%	85.79%	90.49%
199 x 199 x 3	95.21%	85.18%	83.12%	84.13%	84.38%	86.13%	81.20%	89.59%
99 x 99 x 3	95.18%	84.06%	83.01%	83.99%	84.04%	85.71%	81.01%	89.42%
399 x 399 x 3	95.39%	87.77%	88.59%	87.99%	88.97%	88.03%	85.86%	90.57%
499 x 499 x 3	95.45%	87.82%	88.64%	88.04%	89.08%	88.15%	85.94%	90.64%
599 x 599 x 3	95.59%	87.86%	88.72%	88.09%	89.15%	88.21%	85.99%	90.76%

**Fig 12:**
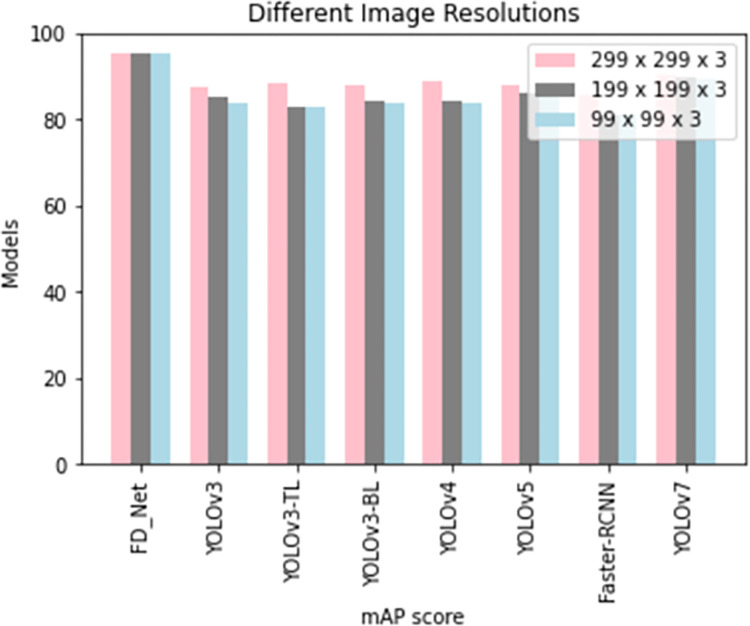
mAP score comparison between the different sizes of images.

After analyzing the outcomes of the experiments, it became clear that the dimensions of the input images had a considerable impact on the performance of FD_Net, YOLOv3, YOLOv3-TL, YOLOv3-BL, YOLOv4, YOLOv5, Faster-RCNN, and YOLOv7. Aside from that, the detection performance is also impacted by the image size. For example, the detection performance improved when the image size was increased, indicating that the larger the image size, the better the detection performance. When we increase the input image size the accuracy of the proposed model and other YOLO models is also increased as shown in Table 5. Similarly, we decrease the image size to 199 x 199 x 3 and 99 x 99 x 3 the proposed model achieved the mAP of 95.21% and 95.18% respectively. The detailed results are graphically presented in Fig 12.

From Table 5, it is concluded that our proposed model achieves significant results as compared to the other seven YOLO versions. Our proposed model is also suitable for the small size of input images used for the detection of the fish species. A comprehensive analysis of nine fish species classes mAP% as it appears in images of varying scales is presented in Table 5.

#### Analysis of the detection time

**Fig 13:**
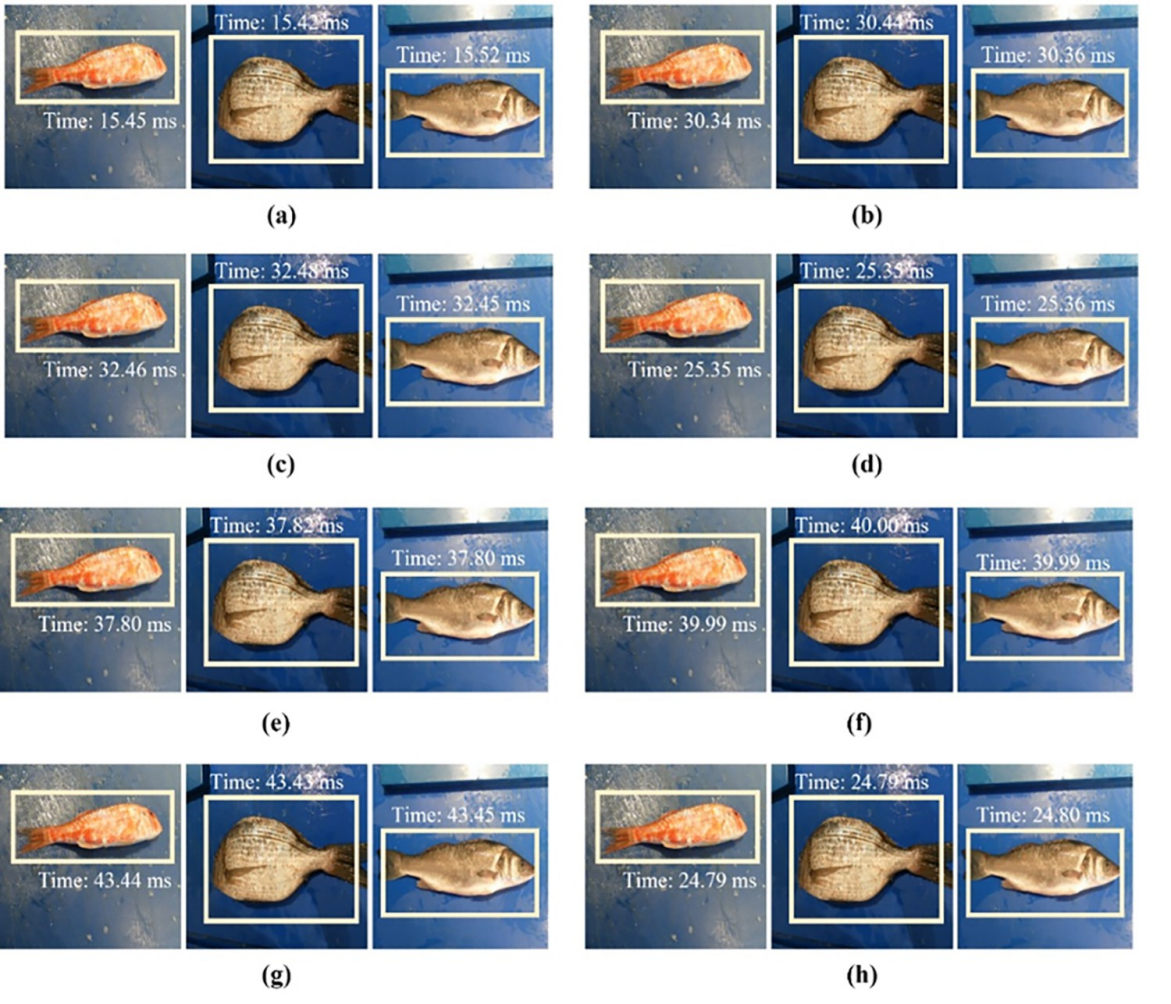
Fish detection time; a) FD_Net, b) YOLOv3, c) YOLOv3-TL, d) YOLOv3-BL, e) YOLOv4, f) YOLOv5, g) Faster-RCNN, and h) YOLOv7 models.

The detection time analysis calculated using images was carried out using a variety of resolutions. The FD_Net, YOLOv3, YOLOv3-TL, YOLOv3-BL, YOLOv4, YOLOv5, Faster-RCNN, and YOLOv7 models were used to analyze each of the images, and the combined scores were used to determine the final result. YOLOv3-BL has a parameter quantity of 67 million, which is double the size of the FD_Net model parameter quantity, which is approximately 32 million (see Table 3). Additionally, YOLOv3-TL, YOLOv4, YOLOv5, and YOLOv7 have 45 million, 72 million, 58 million, and 60 million respectively. The detection speed of FD_Net is much quicker than that of YOLOv3-TL, YOLOv4, YOLOv5, and YOLOv7 due to the reduced number of parameters. Because this study acquired deep separable convolution, the number of parameters was cut by between 3 and 4 times. Therefore, the detection speed of YOLOv3, YOLOv3-TL, YOLOv3-BL, YOLOv4, YOLOv5, Faster-RCNN, and YOLOv7 models are slow, whereas the speed of FD_Net is approximately fifty percent faster than that of YOLOv3-BL. The IoU is the intersection of the anticipated box and the ground truth bounding box. The ideal condition for IoU would be closer to 1, which would indicate that there is a complete overlap in the data. If the IoU score is more than the cutoff value of 0.5, then it has the potential to be regarded as an outstanding performance. The GIoU was utilized so that the bounding box regression loss for the training model could be calculated. According to the findings, the FD_Net model is superior to the other models in terms of its capability to extract finer-grained features of small objects. As a direct result of this, the IoU of small and medium-sized objects has been given a boost in terms of their competitiveness. In addition to this, the IoU value of the improved FD_Net model has almost the same value when compared to the seven different detection models. As can be seen in Fig 13, the estimated result revealed that the FD_Net model displayed much better results in terms of detection time when compared to the YOLOv3, YOLOv3-TL, YOLOv3-BL, YOLOv4, YOLOv5, Faster-RCNN, and YOLOv7 models.

Therefore, it is necessary to take into consideration whether the quantity of mAP should be reduced to get a speedier network. The results of a comparison between FD_Net and other models were displayed in Table 6 in terms of frame per second (FPS) and detection time in milliseconds (ms). Additionally, Table 6 displayed the results obtained by using the image resolutions of 299 x 299 x3.

**Table 6:** Detection time and FPS of the FD_Net and other models.

Models	Image Resolution	Detection Time (ms)	FPS
FD_Net	299 x 299 x 3	15.45	48
YOLOv3		30.34	52
YOLOv3-TL		32.46	56
YOLOv3-BL		25.35	58
YOLOv4		37.80	59
YOLOv5		39.99	60
Faster-RCNN		43.44	53
YOLOv7		24.79	45

### Ablation study

In this study, we improved the model by including the BNAM as well as enhanced versions of the DenseNet-169 and Arcface loss function modules. We used the control variable strategy to statistically analyze the experimental data while simultaneously controlling a variable to determine whether or not the updated YOLOV7 model is valid for use with nine different species of fish. During this work, the mAP and FPS values of each model were analyzed and compared with the help of metrics to identify the importance of the improved module to the model. The initial YOLOV7 model is shown in Experiment 1, the BNAM is implemented in Experiment 2, upgraded DenseNet-169 with an Arcface loss function in Experiment 3, and the improved model is shown in Experiment 4. The findings of the experiment are shown in Table 7.

**Table 7:** Integration of the various components of the model such as YOLOv7, BNAM, and DenseNet-169.

Experiment	YOLOv7	BNAM	DenseNet	Image Resolution	mAP (%)	FPS
1	✓	×	×	299 x 299 x 3	77.98%	62
2	✓	×	✓	299 x 299 x 3	79.63%	59
3	×	×	✓	299 x 299 x 3	80.86%	61
4	✓	✓	✓	299 x 299 x 3	95.30%	48

When the results of Experiment 1 and Experiment 2 are compared, it is clear that the incorporation of the BNAM into the model leads to an improvement of 1.06% in the model’s average detection accuracy (mAP), even though the model’s inference speed is slightly slowed down as a result of this modification. This shows that the BNAM makes use of channel attention to create the correlation across channels, therefore suppressing the information about features that are not necessary, and that it makes use of the spatial attention mechanism to extract the target’s position within a given space. The BNAM allows the model to pay more attention to the feature information of the detection target, which improves the quality of the feature mapping and significantly increases the overall accuracy of the model. On the other hand, the BNAM increases the complexity of the model and slows down the inference speed of the network. When the results of Experiment 1 and Experiment 3 are compared, the model inference speed (FPS) is shown to be improved by 0.92 percent. This shows that replacing the ReLU function with the Arcface activation function increases the model inference speed while keeping the same perceptual field. When Experiment 1 is compared to Experiment 4, the model’s average detection accuracy shows a 14.29% improvement. This indicates that the YOLOv7 model, which combines the BNAM and DenseNet-169 in exchange for improved detection accuracy, is superior in terms of overall model performance.

### Comparison with state-of-the-art methods

In this section, the proposed FD_Net model is compared with the state-of-the-art methods in detecting nine different types of fish species. Table 8 compares the mAP score of our suggested classifier, FD_Net, with other most recent methods. Iqbal et al [68] proposed a CNN-based model for two types of fish species classification and they were able to achieve 88.00 mAP scores. Knausgård et al [2] improved the YOLOv3 and combine it with CNN to classify five different types of fish species. They achieved an mAP score of 87.40%. Similarly, Ju et al [103] fine-tuned the pre-trained model AlexNet in classifying the different types of fish and attained the mAP of 89.78%. Our proposed FD_Net achieved a 95.30% of mAP score in classifying nine different types of fish species. The results reveal that the proposed FD_Net model produced superior results as compared to state-of-the-art methods in detecting fish.

**Table 8:** Proposed FD_Net model comparison with state-of-the-art methods.

Ref	Year	Models	Fish Classes	mAP
Kaya et al [72]	2023	CNN	5	90.39%
Ren et al [73]	2023	CNN and SVM	4	92.43%
Iqbal et al [68]	2022	CNN	2	88.00%
Knausgård et al [2]	2022	YOLOv3 + CNN	5	87.40%
Li et al [97]	2022	FFRNET	2	90.00%
Ju et al [98]	2020	AlexNet	4	89.78%
Tarling et al [7]	2022	ResNet-50	4	90.79%
Yeh et al [11]	2021	CNN	5	91.29%
Ours	-	FD_Net	9	95.30%

## Conclusion and future work

Deep neural networks give a solution to the problem of reliably recognizing fish and other aquatic species of interest, which was previously a difficult task. This research makes use of fish species analyses and substantial data collection to demonstrate the efficacy of the deep neural network approach in deep oceans. This paper proposes a detection module that enhances the YOLOv7 object identification approach by making it lighter and more accurate. The improvement is achieved by improving the feature extraction network and adding depthwise separable convolution in BNAM. In the fish species identification module, the FD_Net method is presented, and DenseNet-169 is improved by the addition of the BNAM attention module as well as the presentation of BAN, pooling layers, a loss function, and dilated convolution. The improved version of DenseNet-169 is utilized as a network for feature extraction. Arcface Loss is used as a loss function, which not only increases the compactness within a class but also assures the separability across classes. The combination of detection and identification algorithms has a beneficial effect, as it permits the realization of a varied range of underwater fish species. The FD_Net, YOLOv3, YOLOv3-TL, YOLOv3-BL, YOLOv4, YOLOv5, Faster-RCNN, and YOLOv7 models are the ones responsible for carrying out the fish detection. The FD_Net model’s detection speed is exceptionally quick because it has fewer parameters than other models. The findings demonstrate that the FD_Net model is superior to the other models in terms of its capacity to extract finer-grained features of small objects. As a consequence of this, the IoU of small and medium-sized images has significantly improved in a competitive sense. The FPS, detection accuracy, and mAP for each class are evaluated in this study. The FD_Net model obtain mAP scores of 95.30% when applied to the testing dataset. Following the completion of this study concluded that FD_Net detection time, accuracy, and detection speed have all improved in comparison to other competing approaches. The limitation of this study is that the proposed FD_Net model is not suitable for classifying underwater species other than fish. Future work will involve further exploration of network models for underwater target recognition with the goals of increasing recognition accuracy and speed, broadening the dataset so that the models can be applied to more scenarios with varying conditions, and promoting the use of vision transformers in such scenarios.

## References

[1] Zhao Shili, Zhang Song, Liu Jincun, Wang He, Zhu Jia, et al. "Application of machine learning in intelligent fish aquaculture: A review." *Aquaculture* 540 2021 pp: 736724.

[2] Knausgård Kristian Muri, Wiklund Arne, Tonje Knutsen Sørdalen Kim Tallaksen Halvorsen, Alf Ring Kleiven, et al. "Temperate fish detection and classification: a deep learning based approach." Applied Intelligence 52, no. 6 2022 pp: 6988–7001.

[3] Yang Xinting, Zhang Song, Liu Jintao, Gao Qinfeng, Dong Shuanglin, et al. "Deep learning for smart fish farming: applications, opportunities and challenges." Reviews in Aquaculture 13, no. 1 2021, pp: 66–90.

[4] Hu Jun, Zhao Dandan, Zhang Yanfeng, Zhou Chengquan, and Chen Wenxuan. "Real-time nondestructive fish behavior detecting in mixed polyculture system using deep-learning and low-cost devices." Expert Systems with Applications 178, 2021 pp: 115051.

[5] Ahmed Md Shoaib, Tanjim Taharat Aurpa, and Md Abul Kalam Azad. "Fish disease detection using image based machine learning technique in aquaculture." Journal of King Saud University-Computer and Information Sciences 34, no. 8 2022 pp: 5170–5182.

[6] Yang Ling, Liu Yeqi, Yu Huihui, Fang Xiaomin, Song Lihua, et al. "Computer vision models in intelligent aquaculture with emphasis on fish detection and behavior analysis: a review." Archives of Computational Methods in Engineering 28, no. 4 2021 pp: 2785–2816.

[7] Tarling Penny, Cantor Mauricio, Albert Clapés, and Sergio Escalera. "Deep learning with self-supervision and uncertainty regularization to count fish in underwater images." PloS one 17, no. 5 2022 pp: e0267759. doi: 10.1371/journal.pone.0267759 35507631 PMC9067705

[8] Bailey Jennifer L., and Sigrid Sandve Eggereide. "Mapping actors and arguments in the Norwegian aquaculture debate." Marine Policy 115 2020 pp: 103898.

[9] Mishachandar B., and Vairamuthu S. "Diverse ocean noise classification using deep learning." Applied Acoustics 181 2021 pp: 108141.

[10] Petrellis Nikos. "Measurement of fish morphological features through image processing and deep learning techniques." Applied Sciences 11, no. 10 2021: 4416.

[11] Yeh Chia-Hung, Lin Chu-Han, Kang Li-Wei, Huang Chih-Hsiang, Lin Min-Hui, et al. "Lightweight deep neural network for joint learning of underwater object detection and color conversion." IEEE Transactions on Neural Networks and Learning Systems 2021. 10.1109/TNNLS.2021.3072414 33900925

[12] Politikos Dimitris V., Fakiris Elias, Davvetas Athanasios, Klampanos Iraklis A., and Papatheodorou George. "Automatic detection of seafloor marine litter using towed camera images and deep learning." Marine Pollution Bulletin 164 2021 pp: 111974. doi: 10.1016/j.marpolbul.2021.111974 33485020

[13] Lou Ranran, Lv Zhihan, Dang Shuping, Su Tianyun, and Li Xinfang. "Application of machine learning in ocean data." Multimedia Systems 2021 pp: 1–10.

[14] Sokolova Maria, Adrià Mompó Alepuz Fletcher Thompson, Mariani Patrizio, Galeazzi Roberto, et al. "A Deep Learning Approach to Assist Sustainability of Demersal Trawling Operations." Sustainability 13, no. 22 2021 pp: 12362.

[15] Salim Nareen OM, Subhi RM Zeebaree Mohammed AM Sadeeq, A. H Radie, Hanan M Shukur, et al. "Study for Food Recognition System Using Deep Learning." In Journal of Physics: Conference Series 2021, vol. 1963, no. 1, p. 012014. IOP Publishing.

[16] Zhao Zhenxi, Liu Yang, Sun Xudong, Liu Jintao, Yang Xinting, et al. "Composited FishNet: Fish detection and species recognition from low-quality underwater videos." IEEE Transactions on Image Processing 30 (2021): 4719–4734. doi: 10.1109/TIP.2021.3074738 33905330

[17] Balk Helge. "Development of hydroacoustic methods for fish detection in shallow water." Faculty of Mathematics and Natural Science, University of Oslo 2001.

[18] Bermejo Sergio. "Fish age classification based on length, weight, sex and otolith morphological features." Fisheries Research 84, no. 2 2007,pp: 270–274.

[19] Boom, Bastiaan J., Phoenix X. Huang, Jiyin He, and Robert B. Fisher. "Supporting ground-truth annotation of image datasets using clustering." In Proceedings of the 21st International Conference on Pattern Recognition (ICPR2012), pp. 1542–1545. IEEE, 2012.

[20] Clark, H. L. "New seafloor observatory networks in support of ocean science research." In MTS/IEEE Oceans 2001. An Ocean Odyssey. Conference Proceedings (IEEE Cat. No. 01CH37295), vol. 1, pp. 245–250. IEEE, 2001.

[21] Xu Wenwei, and Matzner Shari. "Underwater fish detection using deep learning for water power applications." In 2018 International conference on computational science and computational intelligence (CSCI), pp. 313–318. IEEE, 2018.

[22] Wang H., ZeZXeZBePJ Lei X. Zhang B. Zhou, and J Peng. "Machine learning basics." Deep Learn 2016 pp: 98–164.

[23] He, Kaiming, Xiangyu Zhang, Shaoqing Ren, and Jian Sun. "Deep residual learning for image recognition." In Proceedings of the IEEE conference on computer vision and pattern recognition, pp. 770–778. 2016.

[24] Ioffe Sergey, and Szegedy Christian. "Batch normalization: Accelerating deep network training by reducing internal covariate shift." In International conference on machine learning, pp. 448–456. PMLR, 2015.

[25] Krizhevsky Alex, Sutskever Ilya, and Hinton Geoffrey E. "Imagenet classification with deep convolutional neural networks." Communications of the ACM 60, no. 6 2017, pp: 84–90.

[26] Cui Suxia, Zhou Yu, Wang Yonghui, and Zhai Lujun. "Fish detection using deep learning." *Applied Computational Intelligence and Soft Computing* 2020 (2020).

[27] Khan Wisal, Raj Kislay, Kumar Teerath, Roy Arunabha M., and Luo Bin. "Introducing urdu digits dataset with demonstration of an efficient and robust noisy decoder-based pseudo example generator." *Symmetry* 14, no. 10 2022 pp: 1976.

[28] Xu Wenwei, and Matzner Shari. "Underwater fish detection using deep learning for water power applications." In *2018 International conference on computational science and computational intelligence (CSCI)*, pp. 313–318. IEEE, 2018.

[29] Knausgård Kristian Muri, Wiklund Arne, Tonje Knutsen Sørdalen Kim Tallaksen Halvorsen, Alf Ring Kleiven, et al. "Temperate fish detection and classification: a deep learning based approach." *Applied Intelligence* 52, no. 6 (2022): 6988–7001.

[30] Alshdaifat Nawaf Farhan Funkur, Abdullah Zawawi Talib, and Mohd Azam Osman. "Improved deep learning framework for fish segmentation in underwater videos." *Ecological Informatics* 59 (2020): 101121.

[31] Jabeen Kiran, Muhammad Attique Khan Jamel Balili, Alhaisoni Majed, Nouf Abdullah Almujally, et al. "BC2NetRF: Breast Cancer Classification from Mammogram Images Using Enhanced Deep Learning Features and Equilibrium-Jaya Controlled Regula Falsi-Based Features Selection." Diagnostics 13, no. 7 (2023): 1238. doi: 10.3390/diagnostics13071238 37046456 PMC10093018

[32] Villon Sébastien, Chaumont Marc, Subsol Gérard, Sébastien Villéger Thomas Claverie, et al. "Coral reef fish detection and recognition in underwater videos by supervised machine learning: Comparison between Deep Learning and HOG+ SVM methods." In *International Conference on Advanced Concepts for Intelligent Vision Systems*, pp. 160–171. Springer, Cham, 2016.

[33] Ashraf Mahmood & Abrar Mohammad & Qadeer, Nauman Alshdadi, Abdulrahman Sabbah, Thabit Khan, et al. (2023). A Convolutional Neural Network Model for Wheat Crop Disease Prediction. 75. 3867–3882. doi: 10.32604/cmc.2023.035498

[34] Khan Muhammad Attique, Arshad Habiba, Wazir Zada Khan Majed Alhaisoni, Tariq Usman, et al. "HGRBOL2: human gait recognition for biometric application using Bayesian optimization and extreme learning machine." Future Generation Computer Systems 143 (2023): 337–348.

[35] Yang Ling, Liu Yeqi, Yu Huihui, Fang Xiaomin, Song Lihua, et al. "Computer vision models in intelligent aquaculture with emphasis on fish detection and behavior analysis: a review." *Archives of Computational Methods in Engineering* 28, no. 4 (2021): 2785–2816.

[36] Li Daoliang, and Du Ling. "Recent advances of deep learning algorithms for aquacultural machine vision systems with emphasis on fish." *Artificial Intelligence Review* 55, no. 5 (2022): 4077–4116.

[37] Rehman Samra, Muhammad Attique Khan Majed Alhaisoni, Armghan Ammar, Tariq Usman, et al. "A Framework of Deep Optimal Features Selection for Apple Leaf Diseases Recognition." CMC-COMPUTERS MATERIALS & CONTINUA 75, no. 1 (2023): 697–714.

[38] Rehman Samra, Muhammad Attique Khan Majed Alhaisoni, Armghan Ammar, Alenezi Fayadh, et al. "Fruit Leaf Diseases Classification: A Hierarchical Deep Learning Framework." CMC-COMPUTERS MATERIALS & CONTINUA 75, no. 1 (2023): 1179–1194.

[39] Larsen Rasmus, Olafsdottir Hildur, and Bjarne Kjær Ersbøll. "Shape and texture based classification of fish species." In Scandinavian Conference on Image Analysis, pp. 745–749. Springer, Berlin, Heidelberg, 2009.

[40] Naeem Ahmad, Anees Tayyaba, Khawaja Tehseen Ahmed Rizwan Ali Naqvi, Ahmad Shabir, et al. "Deep learned vectors’ formation using auto-correlation, scaling, and derivations with CNN for complex and huge image retrieval." *Complex & Intelligent Systems* (2022): 1–23.

[41] Lantsova Ekaterina. "Automatic recognition of fish from video sequences." 2015.

[42] LeCun Yann, Bengio Yoshua, and Hinton Geoffrey. "Deep learning." nature 521, no. 7553 2015,pp: 436–444. doi: 10.1038/nature14539 26017442

[43] Ulucan Oguzhan, Karakaya Diclehan, and Turkan Mehmet. "A Large-Scale Dataset for Fish Segmentation and Classification." In 2020 Innovations in Intelligent Systems and Applications Conference (ASYU), pp. 1–5. IEEE, 2020.

[44] Spampinato, Concetto, Daniela Giordano, Roberto Di Salvo, Yun-Heh Jessica Chen-Burger, Robert Bob Fisher, et al. "Automatic fish classification for underwater species behavior understanding." In Proceedings of the first ACM international workshop on Analysis and retrieval of tracked events and motion in imagery streams, pp. 45–50. 2010.

[45] Huang Phoenix X., Boom Bastiaan J., and Fisher Robert B. "Underwater live fish recognition using a balance-guaranteed optimized tree." In Asian Conference on Computer Vision, pp. 422–433. Springer, Berlin, Heidelberg, 2012.

[46] Vieira Manuel, Fonseca Paulo J., M. Clara P. Amorim, and Carlos JC Teixeira. "Call recognition and individual identification of fish vocalizations based on automatic speech recognition: An example with the Lusitanian toadfish." The Journal of the Acoustical Society of America 138, no. 6 2015, pp: 3941–3950. doi: 10.1121/1.4936858 26723348

[47] Monczak Agnieszka, Ji Yiming, Soueidan Jamileh, and Montie Eric W. "Automatic detection, classification, and quantification of sciaenid fish calls in an estuarine soundscape in the Southeast United States." PloS one 14, no. 1 2019 pp: e0209914. doi: 10.1371/journal.pone.0209914 30650120 PMC6334970

[48] Harakawa Ryosuke, Ogawa Takahiro, Haseyama Miki, and Akamatsu Tomonari. "Automatic detection of fish sounds based on multi-stage classification including logistic regression via adaptive feature weighting." The Journal of the Acoustical Society of America 144, no. 5 2018 pp: 2709–2718. doi: 10.1121/1.5067373 30522274

[49] Noda Juan J., Travieso Carlos M., and David Sánchez-Rodríguez. "Automatic taxonomic classification of fish based on their acoustic signals." Applied Sciences 6, no. 12 2016 pp 443.

[50] Lin Tzu-Hao, Tsao Yu, and Akamatsu Tomonari. "Comparison of passive acoustic soniferous fish monitoring with supervised and unsupervised approaches." The Journal of the Acoustical Society of America 143, no. 4 2018 pp: EL278–EL284. doi: 10.1121/1.5034169 29716292

[51] Ruiz-Blais, Sebastian, Arturo Camacho, and Mario R. Rivera-Chavarria. "Sound-based automatic neotropical sciaenid fishes identification: Cynoscion jamaicensis." In Proceedings of Meetings on Acoustics 167ASA, vol. 21, no. 1, p. 010001. Acoustical Society of America, 2014.

[52] Ricci Shannon W., Bohnenstiehl DelWayne R., Eggleston David B., M. Lisa Kellogg, and R. Patrick Lyon. "Oyster toadfish (Opsanus tau) boatwhistle call detection and patterns within a large-scale oyster restoration site." PloS one 12, no. 8, 2017 pp: e0182757. doi: 10.1371/journal.pone.0182757 28792543 PMC5549733

[53] Salman Ahmad, Jalal Ahsan, Shafait Faisal, Mian Ajmal, Shortis Mark, et al. "Fish species classification in unconstrained underwater environments based on deep learning." Limnology and Oceanography: Methods 14, no. 9, 2016 pp: 570–585.

[54] Qin Hongwei, Li Xiu, Liang Jian, Peng Yigang, and Zhang Changshui. "DeepFish: Accurate underwater live fish recognition with a deep architecture." Neurocomputing 187 2016 pp: 49–58.

[55] Chan Tsung-Han, Jia Kui, Gao Shenghua, Lu Jiwen, Zeng Zinan, et al. "PCANet: A simple deep learning baseline for image classification?." IEEE transactions on image processing 24, no. 12 2015 pp: 5017–5032. doi: 10.1109/TIP.2015.2475625 26340772

[56] Lin Min, Chen Qiang, and Yan Shuicheng. "Network in network." arXiv preprint arXiv:1312.4400 (2013).

[57] Sun Xin, Shi Junyu, Dong Junyu, and Wang Xinhua. "Fish recognition from low-resolution underwater images." In 2016 9th International Congress on Image and Signal Processing, BioMedical Engineering and Informatics (CISP-BMEI), pp. 471–476. IEEE, 2016.

[58] Zhang Dian, Noel E O’Conner, Andre J Simpson, Chunjie Cao, Suzanne Little, et al. "Coastal fisheries resource monitoring through A deep learning-based underwater video analysis." Estuarine, Coastal and Shelf Science 269, 2022, pp: 107815.

[59] Jäger Jonas, Rodner Erik, Denzler Joachim, Wolff Viviane, and Klaus Fricke-Neuderth. "SeaCLEF 2016: Object Proposal Classification for Fish Detection in Underwater Videos." In CLEF (working notes), pp. 481–489. 2016.

[60] Zhang Zhixue, Du Xiujuan, Jin Long, Wang Shuqiao, Wang Lijuan, et al. "Large-scale underwater fish recognition via deep adversarial learning." Knowledge and Information Systems 64, no. 2, 2022 pp: 353–379.

[61] Pang Jian, Liu Weifeng, Liu Baodi, Tao Dapeng, Zhang Kai, et al. "Interference Distillation for Underwater Fish Recognition." In Asian Conference on Pattern Recognition, pp. 62–74. Springer, Cham, 2022.

[62] Wang He, Zhang Song, Zhao Shili, Wang Qi, Li Daoliang, et al. "Real-time detection and tracking of fish abnormal behavior based on improved YOLOV5 and SiamRPN++." Computers and Electronics in Agriculture 192, 2021 pp: 106512.

[63] Labuguen R. T., Volante E. J. P., Causo A., Bayot R., Peren G., Macaraig R. M., et al. "Automated fish fry counting and schooling behavior analysis using computer vision." In 2012 IEEE 8th International Colloquium on Signal Processing and its Applications, pp. 255–260. IEEE, 2012.

[64] Toh Y. H., Ng T. M., and Liew B. K. "Automated fish counting using image processing." In 2009 international conference on computational intelligence and software engineering, pp. 1–5. IEEE, 2009.

[65] Fabic J. N., Turla I. E., Capacillo J. A., David L. T., and Naval P. C. "Fish population estimation and species classification from underwater video sequences using blob counting and shape analysis." In 2013 IEEE international underwater technology symposium (UT), pp. 1–6. IEEE, 2013.

[66] Khai Hong, Teh Siti Norul Huda Sheikh Abdullah, Mohammad Kamrul Hasan, and Ahmad Tarmizi. "Underwater Fish Detection and Counting Using Mask Regional Convolutional Neural Network." Water 14, no. 2 2022 pp: 222.

[67] Tamou Ben, Abdelouahid Abdesslam Benzinou, and Nasreddine Kamal. "Live Fish Species Classification in Underwater Images by Using Convolutional Neural Networks Based on Incremental Learning with Knowledge Distillation Loss." Machine Learning and Knowledge Extraction 4, no. 3 2022 pp: 753–767.

[68] Iqbal Usama, Li Daoliang, and Akhter Muhammad. "Intelligent Diagnosis of Fish Behavior Using Deep Learning Method." Fishes 7, no. 4 2022 pp: 201.

[69] Roy Arunabha M., Bose Rikhi, and Bhaduri Jayabrata. "A fast accurate fine-grain object detection model based on YOLOv4 deep neural network." Neural Computing and Applications 34, no. 5 2022 pp: 3895–3921.

[70] Roy Arunabha M., Bhaduri Jayabrata, Kumar Teerath, and Raj Kislay. "WilDect-YOLO: An efficient and robust computer vision-based accurate object localization model for automated endangered wildlife detection." *Ecological Informatics* 2022 pp: 101919.

[71] Roy Arunabha M., and Bhaduri Jayabrata. "Real-time growth stage detection model for high degree of occultation using DenseNet-fused YOLOv4." *Computers and Electronics in Agriculture* 193 2022 pp: 106694.

[72] Kaya V. O. L. K. A. N., İsmail Akgül, and Ö. Z. G. E Zencir Tanır. "IsVoNet8: A Proposed Deep Learning Model for Classification of Some Fish Species." *JOURNAL OF AGRICULTURAL SCIENCES* 29, no. 1 (2023).

[73] Ren Lihui, Tian Ye, Yang Xiaoying, Wang Qi, Wang Leshan, et al. "Rapid identification of fish species by laser-induced breakdown spectroscopy and Raman spectroscopy coupled with machine learning methods." *Food Chemistry* 400 (2023): 134043. doi: 10.1016/j.foodchem.2022.134043 36058043

[74] Francescangeli Marco, Marini Simone, Enoc Martínez Joaquín Del Río, Toma Daniel M., et al. "Image dataset for benchmarking automated fish detection and classification algorithms." *Scientific data* 10, no. 1 (2023): 5. doi: 10.1038/s41597-022-01906-1 36596792 PMC9810604

[75] Abangan Alexa, Kopp Dorothee, and Faillettaz Robin. "Artificial intelligence for fish behavior recognition may unlock fishing gear selectivity." *Frontiers in Marine Science* 10 (2023).

[76] Rachman, F., M. N. S. Akbar, and E. Putera. "Fish Disease Detection of Epizootic Ulcerative Syndrome Using Deep Learning Image Processing Technique." In *Proceedings International Conference on Fisheries and Aquaculture*, vol. 8, no. 1, pp. 23–34. 2023.

[77] Long, Jonathan, Evan Shelhamer, and Trevor Darrell. "Fully convolutional networks for semantic segmentation." In Proceedings of the IEEE conference on computer vision and pattern recognition, pp. 3431–3440. 2015. 10.1109/TPAMI.2016.2572683 27244717

[78] Macdougall Doug. Endless novelties of extraordinary interest: The voyage of HMS Challenger and the birth of modern oceanography. Yale University Press, 2019.

[79] Nian Rui, Liu Fang, and He Bo. "An early underwater artificial vision model in ocean investigations via independent component analysis." Sensors 13, no. 7 2013 pp: 9104–9131. doi: 10.3390/s130709104 23863855 PMC3758639

[80] Ogunlana S. O., Olabode O., Oluwadare S. A. A., and Iwasokun G. B. "Fish classification using support vector machine." African Journal of Computing & ICT 8, no. 2 2015 pp: 75–82.

[81] Redmon Joseph, and Farhadi Ali. "Yolov3: An incremental improvement." arXiv preprint arXiv:1804.02767 (2018).

[82] Redmon, Joseph, and Ali Farhadi. "YOLO9000: better, faster, stronger." In Proceedings of the IEEE conference on computer vision and pattern recognition, pp. 7263–7271. 2017.Rathi, D., Jain, S., & Indu, S. (2017, December). Underwater fish species classification using convolutional neural network and deep learning. In 2017 Ninth international conference on advances in pattern recognition (ICAPR) (pp. 1–6). IEEE.

[83] Reithaug Adrian. "Employing Deep Learning for Fish Recognition." Master’s thesis, The University of Bergen, 2018.

[84] Ren Shaoqing, He Kaiming, Girshick Ross, and Sun Jian. "Faster r-cnn: Towards real-time object detection with region proposal networks." Advances in neural information processing systems 28, 2015. doi: 10.1109/TPAMI.2016.2577031 27295650

[85] Rezatofighi, Hamid, Nathan Tsoi, JunYoung Gwak, Amir Sadeghian, Ian Reid, et al. "Generalized intersection over union: A metric and a loss for bounding box regression." In Proceedings of the IEEE/CVF conference on computer vision and pattern recognition, pp. 658–666. 2019.

[86] Sandler, Mark, Andrew Howard, Menglong Zhu, Andrey Zhmoginov, and Liang-Chieh Chen. "Mobilenetv2: Inverted residuals and linear bottlenecks." In Proceedings of the IEEE conference on computer vision and pattern recognition, pp. 4510–4520. 2018.

[87] Simonyan Karen, and Zisserman Andrew. "Very deep convolutional networks for large-scale image recognition." arXiv preprint arXiv:1409.1556 (2014).

[88] Welcomme Robin L. "An overview of global catch statistics for inland fish." ICES Journal of Marine Science 68, no. 8 2011 pp: 1751–1756.

[89] Xu Kelvin, Ba Jimmy, Kiros Ryan, Cho Kyunghyun, Courville Aaron, et al. "Show, attend and tell: Neural image caption generation with visual attention." In International conference on machine learning, pp. 2048–2057. PMLR, 2015.

[90] Mannocci Laura, Baidai Yannick, Forget Fabien, Mariana Travassos Tolotti Laurent Dagorn, et al. "Machine learning to detect bycatch risk: Novel application to echosounder buoys data in tuna purse seine fisheries." Biological Conservation 255 2021 pp: 109004.

[91] Ryazanov Igor, Nylund Amanda T., Basu Debabrota, Ida-Maja Hassellöv, and Alexander Schliep. "Deep learning for deep waters: an expert-in-the-loop machine learning framework for marine sciences." Journal of Marine Science and Engineering 9, no. 2 2021 pp: 169.

[92] Rosales Marife A., Maria Gemel B Palconit, Vincent Jan D Almero, Ronnie S Concepcion, Jo-Ann V Magsumbol, et al. "Faster R-CNN based Fish Detector for Smart Aquaculture System." In 2021 IEEE 13th International Conference on Humanoid, Nanotechnology, Information Technology, Communication and Control, Environment, and Management (HNICEM), pp. 1–6. IEEE, 2021.

[93] Schwartz Shawn T., and Alfaro Michael E. "Sashimi: A toolkit for facilitating high‐throughput organismal image segmentation using deep learning." Methods in Ecology and Evolution 12, no. 12 2021 pp: 2341–2354.

[94] Xu Xiaoling, Li Wensheng, and Duan Qingling. "Transfer learning and SE-ResNet152 networks-based for small-scale unbalanced fish species identification." Computers and Electronics in Agriculture 180 2021 pp: 105878.

[95] Bargelloni Luca, Tassiello Oronzo, Babbucci Massimiliano, Ferraresso Serena, Franch Rafaella, et al. "Data imputation and machine learning improve association analysis and genomic prediction for resistance to fish photobacteriosis in the gilthead sea bream." Aquaculture Reports 20 2021 pp: 100661.

[96] Xu Wenwei, and Matzner Shari. "Underwater fish detection using deep learning for water power applications." In 2018 International conference on computational science and computational intelligence (CSCI), pp. 313–318. IEEE, 2018.

[97] Ramachandran Prajit, Zoph Barret, and Le Quoc V. "Searching for activation functions." *arXiv preprint arXiv*:*1710*.*05941* (2017).

[98] Villon Sébastien, Mouillot David, Chaumont Marc, Darling Emily S., Subsol Gérard, et al. "A deep learning method for accurate and fast identification of coral reef fishes in underwater images." Ecological informatics 48 2018 pp: 238–244.

[99] Shahinfar Saleh, Meek Paul, and Falzon Greg. "“How many images do I need?” Understanding how sample size per class affects deep learning model performance metrics for balanced designs in autonomous wildlife monitoring." Ecological Informatics 57 (2020, pp: 101085.

[100] Zhong Ming, Castellote Manuel, Dodhia Rahul, Juan Lavista Ferres Mandy Keogh, et al. "Beluga whale acoustic signal classification using deep learning neural network models." The Journal of the Acoustical Society of America 147, no. 3 2020,pp: 1834–1841. doi: 10.1121/10.0000921 32237822

[101] Hu An, and Razmjooy Navid. "Brain tumor diagnosis based on metaheuristics and deep learning." International Journal of Imaging Systems and Technology 31, no. 2, 2021, pp: 657–669.

[102] Li Danyang, Su Houcheng, Jiang Kailin, Liu Dan, and Duan Xuliang. "Fish Face Identification Based on Rotated Object Detection: Dataset and Exploration." *Fishes* 7, no. 5 (2022): 219.

[103] Ju Zhiyong, and Xue Yongjie. "Fish species recognition using an improved AlexNet model." *Optik* 223 (2020): 165499.

